# Comparative analysis of LJ-4378 and tirzepatide in mouse models of obesity and weight regain

**DOI:** 10.1007/s12272-025-01575-9

**Published:** 2025-11-07

**Authors:** Heeseong Kim, Seung Woo Kim, Cheoljun Choi, SungUg Joo, Gibae Kim, Minjae Kim, Eunsuk Yang, Jangwon Lee, Sukjae Chung, Lak Shin Jeong, Yun-Hee Lee

**Affiliations:** 1 College of Pharmacy and Research Institute of Pharmaceutical Sciences, Seoul National University, 1 Gwanak-ro, Gwanak-gu, Seoul, 08826 Republic of Korea; 2 Future Medicine Company Limited, 54 Chang-eop-ro, Sujeong-gu, Seongnam, Gyeonggi-do 13449 Republic of Korea

**Keywords:** LJ-4378, Tirzepatide, Obesity, Weight regain, Adipose tissue metabolism

## Abstract

We previously developed LJ-4378, a dual ligand for A_2A_ and A_3_ adenosine receptors, as a potential anti-obesity agent. In this study, we compared the anti-obesity effects of LJ-4378 with those of tirzepatide (TZP), a dual agonist of glucagon-like peptide-1 and glucose-dependent insulinotropic polypeptide receptors, and one of the most potent FDA-approved obesity therapeutics. Using a mouse model of diet-induced obesity, we assessed the effects of LJ-4378 and TZP on body weight loss, metabolic parameters, and post-treatment weight rebound. Mice fed a high-fat diet (HFD) for 10 weeks were treated with vehicle, LJ-4378, or TZP for 14 days. Both LJ-4378 and TZP significantly reduced body weight, adipose tissue mass, and abdominal fat volume; improved glucose tolerance; reduced white adipose tissue inflammation; and enhanced energy expenditure. To assess the durability of the treatment effects, drug administration was discontinued after 14 days, and the mice remained on the HFD for an additional 4 weeks. Notably, LJ-4378-treated mice exhibited attenuated body weight regain, stable food intake, persistent metabolic benefits, and sustained enhancement of energy metabolism, compared to TZP-treated mice. These findings highlight LJ-4378 as an anti-obesity agent that functions independently of appetite suppression and may offer superior long-term benefits by limiting post-treatment weight rebound and preserving metabolic improvements.

**Supplementary Information:**

The online version contains supplementary material available at 10.1007/s12272-025-01575-9.

## Introduction

Obesity is a global health issue characterized by excessive adipose tissue accumulation and is closely associated with metabolic disorders such as type 2 diabetes and cardiovascular disease. Although lifestyle and behavioral interventions remain fundamental, their limited long-term effectiveness often necessitates pharmacological approaches (Ng et al. 2014).

Current anti-obesity drugs use diverse mechanisms to induce weight loss and improve metabolic health (Müller et al. 2022). Among these, glucagon-like peptide-1 (GLP-1) receptor agonists have been extensively studied for their effects on satiety, gastric emptying, and glucose regulation (Moore et al. 2023). These agents have demonstrated clinically meaningful weight reduction in obese individuals.

Tirzepatide (TZP), a dual agonist of GLP-1 and glucose-dependent insulinotropic polypeptide (GIP) receptors, has shown enhanced therapeutic efficacy among the GLP-1 agonists (Yao et al. 2024). Clinical studies have reported weight reductions of up to 22.5% over 72 weeks, along with improvements in glycemic control and cardiovascular risk factors (Jastreboff et al. 2022; Sattar et al. 2022). The weight-lowering effects of TZP are mediated through multiple mechanisms: they enhance satiety and reduce food intake (Geisler et al. 2023), while also promoting energy metabolism through GIP receptor-mediated pathways (Regmi et al. 2024; Yu et al. 2025). Specifically, GIPR agonism by TZP has been shown to increase lipid uptake and oxidation in adipocytes, processes associated with elevated thermogenesis, and metabolic activity in the adipose tissue. These data support the use of TZP’s as an effective pharmacological option for obesity and its associated metabolic conditions.

However, maintaining weight long-term remains a major challenge. Weight regain after treatment discontinuation is commonly observed, as many anti-obesity drugs primarily act by reducing caloric intake, and cessation often leads to rapid weight rebound (Müller et al. 2022; Wilding et al. 2022). Thus, novel therapeutics that not only induce weight loss but also sustain metabolic improvements are needed.

LJ-4378 is an investigational compound that acts as an A_2A_ adenosine receptor agonist and A_3_ receptor antagonist. Stimulating A_2A_-mediated adenylyl cyclase activity and blocking A_3_-mediated inhibitory signaling increases intracellular cyclic adenosine monophosphate (cAMP) levels (Im et al. 2021). Elevated cAMP activates protein kinase A (PKA)-dependent pathways, thereby promoting lipolysis and browning of adipocytes. In our preclinical studies, LJ-4378 reduced body weight and enhanced energy expenditure, suggesting its potential role in regulating energy metabolism independent of appetite suppression (Kim et al. 2022). Although these findings are promising, they have not been evaluated against established anti-obesity therapies.

In this study, we compared the anti-obesity efficacy of LJ-4378 and TZP using a diet-induced obesity model and a post-treatment rebound paradigm. TZP was selected as a clinically relevant benchmark because of its proven efficacy and safety. We assessed the effects of LJ-4378 on weight loss, metabolic parameters, and weight maintenance after cessation of treatment. These findings may help guide the development of durable therapeutic strategies for obesity management.

## Materials and methods

### Animals

Animal experiments were conducted in accordance with the guidelines approved by the Institutional Animal Care and Use Committees (IACUC) of Seoul National University (SNU-230113–5-2, SNU-250410–1, and SNU-250811–5). C57BL/6 J male mice and Sprague–Dawley (SD) male rats were raised in a controlled animal facility with a temperature of 22 ± 1 °C and subjected to a 12-h light/12-h dark cycle. Mice were randomly assigned to experimental groups and fed either a normal chow diet (NCD, protein: 24.52% calories, carbohydrates: 63.07% calories, fat: 12.41% calories, 38,057, Purina Lab, Seongnam, Republic of Korea) or a high-fat diet (HFD, protein: 20% kcal, carbohydrate: 20% kcal, fat: 60% kcal, New Brunswick, D12492, Research Diets, NJ, USA) for the specified durations, according to group assignment. Both diet and water were provided ad libitum. SD rats (230–240 g) were provided standard rat chow and had free access to water. Prior to the experiment, the rats underwent a 12-h fasting period.

To evaluate the anti-obesity effect in vivo, mice fed either NCD or HFD for 10 weeks were subsequently treated for up to 4 weeks. To control for injection route-related variables, all mice received both intraperitoneal (I.P.) and subcutaneous (S.C.) injections. The administration were as follows: LJ-4378 treated mice received LJ-4378, which was synthesized in our laboratory at > 95% purity (1 mg/kg/day, I.P.) (Hou et al. 2010; Kim et al. 2022) along with the vehicle for TZP (40 mM Tris–HCl, pH 8, S.C.); TZP treated mice received TZP (Samms et al. 2021; Kim et al. 2022) (LY3298176, MedChemExpress, Monmouth Junction, NJ, USA) (10 nmol/kg/day, S.C.) along with the vehicle for LJ-4378 (0.2% DMSO, I.P.); vehicle control mice received both vehicles (0.2% DMSO, I.P. and 40 mM Tris–HCl, pH 8, S.C.)

To assess post-treatment weight rebound (WR), the mice previously treated with vehicle, LJ-4378, or TZP were maintained on HFD for an additional 4 weeks following treatment cessation. Treatment protocols for the WR groups were identical to those of the HFD groups. In total, nine experimental groups were established: NCD + CTL, NCD + LJ, NCD + TZP; HFD + CTL, HFD + LJ, HFD + TZP; and WR + CTL, WR + LJ, WR + TZP. The volume of oxygen consumed (VO_2_), carbon dioxide produced (VCO_2_), respiratory exchange ratio (RER), energy expenditure, activity, and food intake were measured with PhenoMaster (TSE Systems, Bad Homburg, Germany). Indirect calorimetry was performed under controlled conditions, with a stable ambient temperature of 24 °C and a 12 h light/12-h dark cycle. Mice fed HFD were administered vehicle, LJ-4378, or TZP for 14 days, and the last 2 days of treatment were conducted during indirect calorimetry analysis. The WR model was examined by indirect calorimetry after 2 and 4 weeks of the weight regaining phase.

Additionally, food consumption was measured every 2 days during the 14-day treatment period and for an additional 16 days post-administration.

### In vitro evaluation of CYP enzyme inhibition

Cytochrome 450 (CYP) enzyme inhibition was evaluated using pooled human liver microsomes (Corning, #452117). The assay included the following reagents: ketoconazole (CYP3A4 selective inhibitor, Sigma Aldrich, K1003), Nicotinamide adenine dinucleotide phosphate (NADPH) regeneration system (Promega, V9510), terfenadine as the internal standard (Sigma Aldrich, T9562), and 0.1 M potassium phosphate buffer (pH 7.4, Corning, #451201). Substrates and corresponding metabolites for each CYP isoform were as follows: phenacetin and acetaminophen for CYP1A2 (Sigma Aldrich, #77440 and A7085, respectively), diclofenac and 4'-hydroxydiclofenac for CYP2C9 (Sigma Aldrich, D6899; TRC, H825225), S-mephenytoin and 4'-hydroxymephenytoin for CYP2C19 (Sigma Aldrich, UC175; Santacruz, sc210197), dextromethorphan and dextrorphan for CYP2D6 (Sigma Aldrich, #81091 and UC205), and midazolam and 1'-hydroxymidazolam for CYP3A4 (KFDA; Sigma Aldrich, UC430).

The reaction mixture, containing human liver microsomes (0.25 mg/mL), phosphate buffer, a cocktail of the five CYP substrates, and the test compound LJ-4378 (0 or 10 μM). Samples were preincubated at 37 °C for 5 min, and the reaction was initiated by the addition of the NADPH regeneration system, followed by incubation at 37 °C for 15 min. The reaction was terminated by adding acetonitrile containing the internal standard, and centrifuged at 15,000 rpm for 5 min at 4 °C. The supernatant was analyzed by LC–MS/MS using a Nexera XR system (Shimadzu, Japan) coupled with a TSQ Vantage mass spectrometer (Thermo, USA). Chromatographic separation was performed on a Kinetex C18 column (2.1 × 100 mm, 2.6 μm; Phenomenex, USA) with a gradient elution of water (A) and acetonitrile (B), both containing 0.1% formic acid. Metabolites were quantified in multiple reaction monitoring (MRM) mode with Xcalibur software (version 4.4).

All experiments were performed in duplicate with variability ≤ 15%. The percent activity of each CYP enzyme was calculated based on metabolite formation relative to the no-inhibitor control. Ketoconazole (0.1 μM) was used as a positive control for CYP3A4 inhibition, and the resulting activity (25% ± 10%) was consistent with internal validation criteria.

### In vitro hERG inhibition assay

CHO cells (ATCC #CRL-61) stably expressing hERG potassium channels from Sophion Biosciences were used for this test. The cells were cultured in a humidified incubator with 5% CO_2_ at 37 °C. CHO cells that were at least two days post-plating and more than 75% confluent were used for experiments. Before testing, cells were harvested using TrypLE and resuspended in physiological solution at RT. The physiological and external solutions were prepared at least one month in advance. The intracellular solution was prepared in batches, aliquoted, and stored at 4 °C until use.

Stock solutions of the test compound were prepared in 100% DMSO at 10 mM. These stock solutions were then diluted into the external solution to achieve the final concentrations of 1, 10, and 30 μM. A visual inspection for precipitation was performed before testing. The final DMSO concentration in the extracellular solution did not exceed 0.30%.

The hERG SyncroPatch (384PE) assay was conducted at RT. The setup, prime chip, cell catch and seal, amplifier settings, voltage, and application protocols were controlled using Biomek software (Nanion). A 40 μL addition of vehicle was first applied, followed by a 300-s baseline period. Then, 40 μL of the test compound at the desired concentration was added. Exposure time for each test concentration was no less than 300 s. The entire recording process was subjected to quality control, and wells failing to meet criteria were automatically excluded and retested using PatchControl software.

Each compound was tested at three concentrations (1, 3, and 10 μM), with a minimum of two replicates per concentration.

### Serum biochemistry and cytokine analysis

Serum aspartate aminotransferase (AST), alanine aminotransferase (ALT), blood urea nitrogen (BUN), Creatinine, Creatine kinase MB (CKMB), and lactate dehydrogenase (LDH) levels were analyzed with Fujifilm Dri-Chem 3500 s (Fujifilm, Kanagawa, Japan) according to the manufacturer’s instructions. The slides GOT/AST-P III, GPT/ALT-P III, BUN-P III, CRE-P III, CKMB-P, and LDH-P III were used.

Serum free fatty acid (FFA) levels were quantification using HR Series NEFA-HR (2) (Color Reagent A (#999–34691), Solvent A (#995–34791), Color Reagent B (#991–34891), Solvent B (#993–35191), and NEFA Standard Solution (#276–76491) (FUJIFILM Wako Pure Chemicals, Osaka, Japan), following the manufacturer’s instruction.

The serum TNF-α level was measured using TNF-alpha ELISA MTA00B (R&D system) in accordance with the manufacturer’s protocol.

### Body composition and micro-computer tomography (micro-CT)

Body composition and micro-CT analysis were performed after the treatment with vehicle, LJ-4378, or TZP for the indicated duration in HFD-fed mice and WR mice.

A nuclear magnetic resonance (NMR) technology system with EchoMRI-700 (Echo Medical Systems, Nanded, India) was used to analyze body composition.

To assess the abdominal fat volume, we employed the Quantum GX2 micro-CT imaging system (PerkinElmer, Waltham, MA, USA). Mice were anesthetized with isoflurane and positioned on the micro-CT bed for imaging of the abdominal region, defined from the proximal end of the L1 vertebra to the distal end of the L5 vertebra. Imaging was performed at 90 kV/88 µA with a field of view of 72 mm, a pixel size of 90 µm, and a 2-min acquisition time at standard resolution. Visceral, subcutaneous, and total abdominal fat volumes were quantified using AnalyzeDirect software (ver. 12.0, Overland Park, KS, USA).

### Immunoblot analysis

For protein extraction, we utilized PRO-RERP Protein Extraction Solution (17081, iNtRON Biotechnology, Gyeonggi-do, Korea), supplemented with SIGMAFAST Protease Inhibitor (S8820, Sigma, St. Louis, MO, USA) and PhosSTOP phosphatase inhibitor (#4906837001, Roche, Basel, Switzerland). Protein concentrations were determined using the Pierce BCA protein assay Kit (#23227, Thermo Fisher, Waltham, Massachusetts, USA) by measuring absorbance at 562 nm with a MultiSkan GO spectrophotometer (Thermo Fisher). The quantified proteins were loaded and separated in 12% SDS-PAGE gels and transferred to polyvinylidene difluoride (PVDF) membranes (Bio-Rad, Hercules, CA, USA). Membranes were blocked in 5% bovine serum albumin or skim milk in Tris-buffered saline with 0.1% Tween 20 (TBST) for 1 h, followed by overnight incubation with primary antibodies. The next day, membranes were washed 3 times with TBST and incubated for 1 h with horseradish peroxidase (HRP)-conjugated secondary antibody: Goat anti-rabbit (1:3000, #31460, Thermo Fisher) and Goat anti-Mouse IgG (1:3000, 115–035-174, Jackson ImmunoResearch). After secondary antibody incubation, membranes were washed 3 times with TBST and detected with the Fusion Solo chemiluminescence imaging system (Vilber Lourmat, France). EvolutionCapt (version 17.03) software and the ImageJ (National Institutes of Health, Bethesda, MD, USA) program were used for analysis and to quantify the protein level. The primary antibodies are listed in Table 1.

**Table 1:** Primary antibodies list for immunoblot

Antibody	Host	Manufacturer	Catalog #	Dilution
F4/80	Rabbit	Cell signaling	30325S	1:1000
Phospho-HSL	Rabbit	Cell signaling	45804S	1:1000
HSL	Rabbit	Cell signaling	4107S	1:1000
Phospho-CREB	Rabbit	Cell signaling	9198S	1:1000
CREB	Rabbit	Cell signaling	9197S	1:1000
COXIV	Rabbit	Cell signaling	4850S	1:1000
UCP1	Rabbit	Alpha diagnostic	UCP11-A	1:1000
Total OXPHOS cocktail	Mouse	Abcam	Ab110413	1:1000
α/β-Tubulin	Rabbit	Cell signaling	2148S	1:1000
β-actin	Mouse	Santa Cruz	sc-47778	1:1000

### Real-time quantitative PCR (RT-qPCR)

RT-qPCR was conducted according to the method (Cho et al. 2022). The tissue samples were lysed in TRIzol (#15596018, Thermo Fisher), and the total RNA was extracted. The extracted RNA was synthesized into cDNA using the cDNA Reverse Transcription Kit (Applied Biosystems, Waltham, MA, USA). mRNA levels were quantified using iQ SYBR Green Supermix on the CFX Connect Real-time system (Bio-rad). Relative expression levels of each gene were calculated using the 2ΔΔCt method, with the reference housekeeping gene as follows: Peptidylprolyl isomerase A (*Ppia*) for adipose tissue and glyceraldehyde-3-phosphate dehydrogenase (*Gapdh*) for hypothalamus. Primer sequences used for qPCR are listed in Table 2.

**Table 2:** Primer sequences for qPCR

Gene	Forward 5’-3’		Reverse 5’-3’
*Tnf-α*	GGTGCCTATGTCTCAGCCTCTT		GCCATAGAACTGATGAGAGGGAG
*Emr1*	GTGACTCACCTTGTGGTCCT		CAGACACTCATCAACATCTGCG
*Arg1*	GGCTGGTGTGGTGGCAGAGG		CCTGGCGTGGCCAGAGATGC
*Clec10a*	ATGGGTGGATGGGACCGACTTT		GGAAGGTTCTCTGGCAGACATC
*Chil3*	TACTCACTTCCACAGGAGCAGG		CTCCAGTGTAGCCATCCTTAGG
*Il-10*	CAG TACAGCCGGGAA GACAA		GGCAACCCAAGTAACCCTTA
*Npy*	AGACCCTTCCATGTGGTGAT		GAGATGAGGGTGGAAACTTGGA
*Pomc*	CCATAGATGTGTGGAGCTGGTG		CATCTCCGTTGCCAGGAAACAC
*Ppia*	GTGGTCTTTGGGAAGGTGAA		TTACAGGACATTGCGAGCAG
*Gapdh*	GGAGAGTGTTTCCTCGTCCC		ACTGTGCCGTTGAATTTGCC

### Glucose tolerance test (GTT)

The glucose tolerance test was conducted on mice fasted for 12 h with ad libitum access to water. Mice were intraperitoneally injected with 20% D-( +)-glucose (2 g/kg body weight, #49139, Sigma), and blood glucose levels were measured at each time point using a Gluco Doctor Top meter and strip (Allmedicus, South Korea).

### Histology and Immunohistochemistry analysis

Adipose tissues were fixed in 10% formalin (HT50128, Sigma) for 2 days and made into paraffin slides. The deparaffinized sections were stained with ClearView Staining Hematoxylin (MA010081, BBC Biochemical, Mount Vernon, WA, USA) and Eosin Y Alcoholic (#3610, BBC Biochemical) solution. The crown-like structure formation of gonadal white adipose tissue (gWAT) and triglyceride (TG) accumulation in the liver are observed using hematoxylin and eosin staining on paraffin slides. The images were captured using Nikon Elements (NIS BR Analysis ver 5.10.00).

Immunohistochemistry was performed on deparaffinized gWAT sections using an anti-F4/80 antibody (1:500, MCA497GA, Bio-rad). After overnight incubation with the primary antibody, sections were incubated for 1 h at RT with secondary antibody: Donkey anti-Rat 488 Alexa Fluor™ 488 (1:500, A21208, Invitrogen). Lastly, DAPI (1:1000, D9542, Sigma) was incubated for 4 min at RT to stain nuclei. Images were acquired using a Leica THUNDER Imager 3D (Leica Microsystems, Buffalo Grove, IL, Germany). Image analysis and F4/80 signal quantification were performed using LAS X software (ver. 3.6.0) and ImageJ.

### Liver TG quantification

Hepatic TG content was measured following a protocol (Lee et al. 2024). Briefly, 100 mg of liver tissue was digested overnight at 55 °C in 300 μL of ethanolic KOH solution (Ethanol: 30% KOH = 2:1). The digested sample was then adjusted to a total volume of 1000 μL with 50% ethanol and centrifuged at 1000 rpm for 5 min. The supernatants were transferred to a new tube, and 50% Ethanol was added to bring the final volume to 12,000 μL. The 200 μL of the sample was then mixed with 215 μL of 1 M MgCl_2_, incubated on ice for 10 min, and centrifuged. The supernatant was collected for TG quantification. Triolein level was measured using glycerol reagent (F6428, Sigma) according to the manufacturer’s instructions. Hepatic TG content was expressed as triolein equivalents.

### Plasma protein binding assay and in vitro evaluation of CYP enzyme inhibition

The plasma protein binding of LJ-4378 was evaluated using the Rapid Equilibrium Dialysis (RED) method with 100% plasma from human (Biochemed, BC22089NCP), rat (Innovative, IRT-N), and mouse (Biochemed, 029-APSC-MP), all treated with sodium citrate as an anticoagulant. The RED device (ThermoFisher Scientific, #89809) and reusable base plate (ThermoFisher Scientific, #89811) were used with an 8 K MWCO membrane. Test compounds were added to plasma at a final concentration of 10 μM and dialyzed against phosphate-buffered saline (PBS, pH 7.4; Hyclone, #SH30256.01) at 37 °C for 4 h. After incubation, equal volumes of plasma and PBS samples were mixed with blank PBS and blank plasma, respectively, followed by the addition of acetonitrile containing chlorpropamide (TRC, C424800) as the internal standard. The mixtures were centrifuged at 15,000 rpm for 5 min at 4 °C, and the supernatants were analyzed using LC–MS/MS. Chromatographic separation was performed on a Kinetex C18 column (2.1 × 100 mm, 2.6 μm; Phenomenex) using a gradient elution of water with 0.1% formic acid (A) and acetonitrile with 0.1% formic acid (B), on a Nexera XR system (Shimadzu, Japan) coupled to a TSQ Vantage mass spectrometer (Thermo, USA) operating in MRM mode with Xcalibur 4.4 software. All measurements were performed in duplicate with a deviation of less than 15% between replicates. The free fraction (% Free) was calculated as (concentration in buffer chamber/concentration in plasma chamber) × 100, and the bound fraction (% Bound) was obtained by subtracting % Free from 100. In cases where measurements were unstable, deviations greater than 15% were allowed.

### Plasma stability assay

The plasma stability of LJ-4378 was evaluated using 100% plasma obtained from human (Biochemed, BC22089NCP, sodium citrate), rat (Innovative, IRT-N, sodium citrate), and mouse (Biochemed, 029-APSC-MP, sodium citrate). 10 Μm of LJ-4378 was added to the plasma, and samples were incubated at 37 °C for 0, 30, and 120 min. At each time point, an aliquot of the plasma was removed and mixed with acetonitrile containing the internal standard, chlorpropamide (TRC, C424800). The mixture was vortexed for 5 min and centrifuged at 15,000 rpm for 5 min at 4 °C. The supernatant was injected into the LC–MS/MS system for quantification of the remaining compound. Analysis was performed using a Nexera XR system (Shimadzu, Japan) with a TSQ Vantage mass spectrometer (Thermo, USA). Chromatographic separation was achieved using a Kinetex C18 column (2.1 × 100 mm, 2.6 μm particle size; Phenomenex, USA) with a gradient elution of 0.1% formic acid in water (mobile phase A) and 0.1% formic acid in acetonitrile (mobile phase B). Data acquisition was conducted in MRM mode using Xcalibur software (version 4.4). All experiments were performed in duplicate with a deviation within 15%, and the percentage of compound remaining (% Remaining) at each time point was calculated relative to the non-incubated control sample. For quality control, procaine and enalapril (each at 10 μM) were used as reference compounds and were confirmed to fall within acceptable ranges.

### Pharmacokinetic study in mice

The pharmacokinetics of LJ-4378 were evaluated in male C57BL/6 J mice following a single administration by either the oral (P.O.) or intravenous (I.V.) route. For P.O., LJ-4378 was administered at 10, 20, or 50 mg/kg in a vehicle composed of 0.5 mg/mL *L*-ascorbic acid, 5% DMSO, 20% PEG400, 20% Tween 80, and 55% of 0.1% copovidone. For I.V. administration, a 2 mg/kg dose was delivered via tail vein injection in a vehicle consisting of 5% DMSO, 25% PEG400, and 70% sterile water. Blood samples (200 μL) were collected at 5, 15, and 30 min and 1, 2, 4, 8, 12, and 24 h post-dose for the P.O. groups, and at 2, 5, 15, and 30 min and 1, 2, 4, 8, and 12 h post-dose for the I.V. group. Plasma was separated by centrifugation, and the concentrations of LJ-4378 were determined using a validated LC–MS/MS method. Pharmacokinetic parameters were calculated using non-compartmental analysis with Phoenix WinNonlin (v8.3), and included C_max_, T_max_, AUC_last_, AUC_inf_, t_1/2_, and clearance (Maclean et al. 2011). Oral bioavailability was calculated using dose-normalized AUC_inf_ values.

### *Determination of *in vivo* steady state tissue partition coefficient of LJ-4378 in rats*

In this study, steady state tissue partition coefficients ($K_{P,T}$, i.e., the ratio of tissue to plasma concentrations in steady state) Eq. (1) were determined for LJ-4378 in SD rats. In our preliminary study involving I.V. bolus administration of LJ-4378 to mice, the terminal phase half-life was determined to be approximately 0.29 h. Therefore, to ensure a steady state condition, rats received I.V. infusion (i.e., infusion solution, 5% DMSO in normal saline; delivery rate, 10 µL/min; infusion rate, 0.31 µg/µL; total dose of LJ-4378, 0.837 mg per rat) for 270 min (i.e., the time exceeding five times the half-life). Blood samples (~ 150 µL) were collected from the animal at every 10 min from 240 to 270 min for the confirmation of the steady state of the compound. At completion of the last blood collection, rats were sacrificed by decapitation, and four representative tissues (i.e., gonadal adipose, brain, lung, and liver) were harvested. Tissues were then washed four times with ice-Dulbecco’s phosphate-buffered saline (DPBS), weighed, and homogenized in two volumes of DPBS using an Ultra Turrax homogenizer (IKA, Staufen, Germany). Aliquots (50 µL) of plasma and tissue homogenate were mixed with 200 µL ice-cold acetonitrile containing propranolol (100 ng/mL, internal standard). After vortex mixing for 10 min, the mixture was centrifuged at 16,100 × g for 5 min at 4 °C, and 10 µL of the supernatant was injected directly onto the HPLC–MS/MS system. The concentration of LJ-4378 in the biological sample was quantified on an Agilent 1200 HPLC coupled to an API 3200 mass spectrometer with a Phenomenex Luna C18(2) column (3 µm, 100 Å, 50 × 2 mm). Chromatography was performed under isocratic conditions with 80% acetonitrile containing 0.1% formic acid (mobile phase A) and 20% water containing 0.1% formic acid (mobile phase B) at a flow rate of 0.4 mL/min.

When calculating $K_{P,T}$ for LJ-4378 in SE rats, Eq. (1) was used:

1 $$K_{P,T} = \frac{{C_{T,ss} }}{{C_{p,ss} }}$$

where $C_{T,ss}$ and $C_{p,ss}$ represented the tissue and plasma concentrations at 270 min after the initiation of LJ-4378 infusion. In addition, under the free-drug hypothesis, the $K_{P,T}$ can be expressed as the ratio of the unbound fractions in plasma ($f_{up}$) and tissue ($f_{u,T}$) [Eq. (2)]. Given similar interspecies values of tissue unbound fraction (Poulin and Theil 2000), the rat $K_{P,T}$ values were extrapolated to those in mice and humans using the following Eq. (3):

2 $$K_{P,T} = \frac{{f_{up} }}{{f_{u,T} }}$$

3 $$K_{P,T,extrapolated} = K_{P,T,rat} \times \frac{{f_{up} }}{{f_{up,rat} }}$$

### Statistical analysis

We conducted the statistical analysis using GraphPad Prism 8 (Software Inc., San Diego, CA, USA). Data are presented as mean ± standard errors of the means (SEMs). Statistical significance between two groups was determined using an unpaired two-tailed *t*-test. For comparisons involving more than two groups, one-way or two-way ANOVA was applied, followed by Bonferroni post hoc tests to adjust for multiple comparisons. The specific statistical tests used for each dataset are indicated in the corresponding figure legends.

## Results

### LJ-4378 and TZP treatments reduce body weight and adiposity in HFD-fed mice

Before conducting in vivo preclinical studies, we evaluated the safety profile of LJ-4378. First, we assessed cytochrome 450 (CYP450) inhibition and human ether-à-go-go-related gene (hERG) channel blockade. LJ-4378 exhibited minimal inhibitory activity against the major human CYP isoforms when tested at a concentration of 10 μM. Residual enzymatic activities were maintained at 82.2% (CYP1A2), 95.8% (CYP2C9), 85.3% (CYP2C19), 89.3% (CYP2D6), and 96.9% (CYP3A4), indicating low potential for drug-drug interactions (Table S1). The hERG inhibition assay revealed a half-maximal inhibitory concentration (IC50) value greater than 30 μM, suggesting a low risk of cardiotoxicity. Furthermore, in vivo analyses of hepatic, renal, and cardiac injury markers revealed no evidence of organ toxicity, supporting the favorable safety profile of LJ-4378 (Fig. S1).

**Fig. 1:**
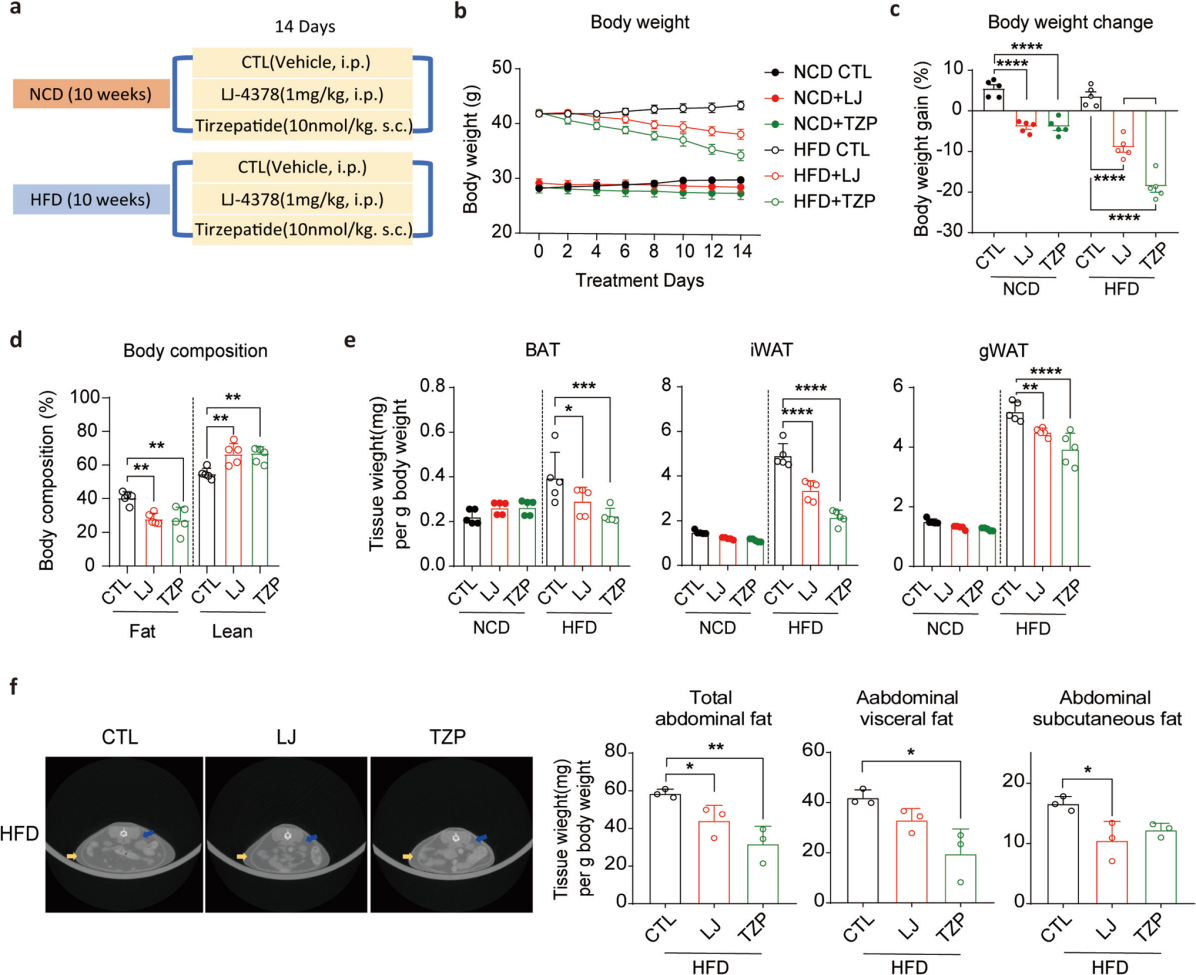
LJ-4378 and TZP reduce body weight and fat mass in mice fed HFD. **a**. Schematic representation of the experimental design for normal chow diet (NCD) and high-fat diet (HFD) groups. Mice received either vehicle [(0.2% DMSO, intraperitoneal (I.P.) and 40 mM Tris–HCl, pH 8, subcutaneous (S.C.)], LJ-4378 (1 mg/kg, I.P.), or tirzepatide (TZP, 10 nmol/kg, S.C.) once daily for 14 days. **b**. Body weight changes over 14 days of vehicle, LJ-4378, or TZP treatment. n = 5. **c**. Body weight gain ratio following vehicle, LJ-4378, or TZP treatment in NCD- or HFD-fed mice. n = 5. Significant effects of diet (*p* < 0.0001) and genotype effects (*p* < 0.0001) were observed. **d**. Percentage of body fat and lean mass in HFD-fed mice treated with vehicle, LJ-4378, or TZP. n = 5. Significant group effects (Fat: *p* = 0.0027, Lean: *p* = 0.0034) were observed. **e**. Adipose tissue weights, including brown adipose tissue (BAT), inguinal white adipose tissue (iWAT), and gonadal white adipose tissue (gWAT), after treatment with LJ-4378 or TZP in NCD- or HFD-fed mice. n = 5. Significant effects of diet (BAT: *p* = 0.0792, iWAT: *p* < 0.0001, gWAT: *p* < 0.0001) and treatment (BAT: *p* = 0.0182, iWAT: *p* < 0.0001, gWAT: *p* < 0.0001) were observed. **f**. Representative micro-CT images and quantification of abdominal fat volumes (total, subcutaneous, and visceral) in HFD-fed mice treated with vehicle, LJ-4378, or TZP. n = 3. (Yellow arrows indicate subcutaneous adipose depot, and blue arrows indicate visceral adipose depot) Significant group effects (Total abdominal fat: *p* = 0.0063, Abdominal visceral fat: *p* = 0.0100, Abdominal subcutaneous fat: *p* = 0.0277) were observed. Statistical significance was determined using two-way ANOVA followed by Bonferroni post hoc test in** c** and **e**, and **a** one-way ANOVA followed by Bonferroni post hoc test in **d** and **f**. Values are presented as mean ± SEM (^∗∗∗∗^*p* < 0.0001, ^∗∗∗^*p* < 0.001, ^∗∗^*p* < 0.01, ^∗^*p* < 0.05)

We compared the anti-obesity effects of LJ-4378 with those of TZP in mice fed a normal chow diet (NCD) or HFD for 10 weeks (Fig. 1a). After HFD-induced obesity, the mice were administered vehicle, LJ-4378, or TZP for 14 days. LJ-4378 treatment resulted in a 9.04% reduction in total body weight, whereas TZP showed a more pronounced reduction of 18.63% compared to the HFD control group (Fig. 1b, c). Although TZP-treated mice exhibited greater weight loss, LJ-4378 and TZP reduced fat mass by 30.7% and 32.4%, respectively, relative to the fat mass of HFD controls (Fig. 1d). Additionally, both compounds significantly reduced inguinal white adipose tissue (iWAT) and gonadal white adipose tissue (gWAT) mass, whereas TZP reduced brown adipose tissue (BAT) mass in HFD-fed mice (Fig. 1e). Micro-computed tomography (micro-CT) analysis further confirmed the reduction in both visceral and subcutaneous abdominal fat following treatment with either compound in HFD-fed mice (Fig. 1f).

Collectively, these findings demonstrated the weight-reducing effects of LJ-4378 and TZP. Notably, despite differences in total weight loss, similar reductions in fat mass suggest that LJ-4378 may preferentially target fat mass while preserving lean tissue. To compare the fat-specific effects of LJ-4378 with those of TZP under equivalent total weight loss, we extended the LJ-4378 treatment until the body weight matched that of the 2-week TZP group. Four weeks of LJ-4378 administration produced a comparable reduction (15.5%) in body weight compared to 2 weeks of TZP treatment (Fig. S2a, b, d). Notably, LJ-4378 exhibited a lower fat ratio (20.1% of the total body weight) compared to 2 weeks of TZP treatment (27.4% of the total body weight) (Fig. S2c, e).

### LJ-4378 and TZP improve glucose tolerance and alleviate adipose tissue inflammation in HFD-fed mice

**Fig. 2:**
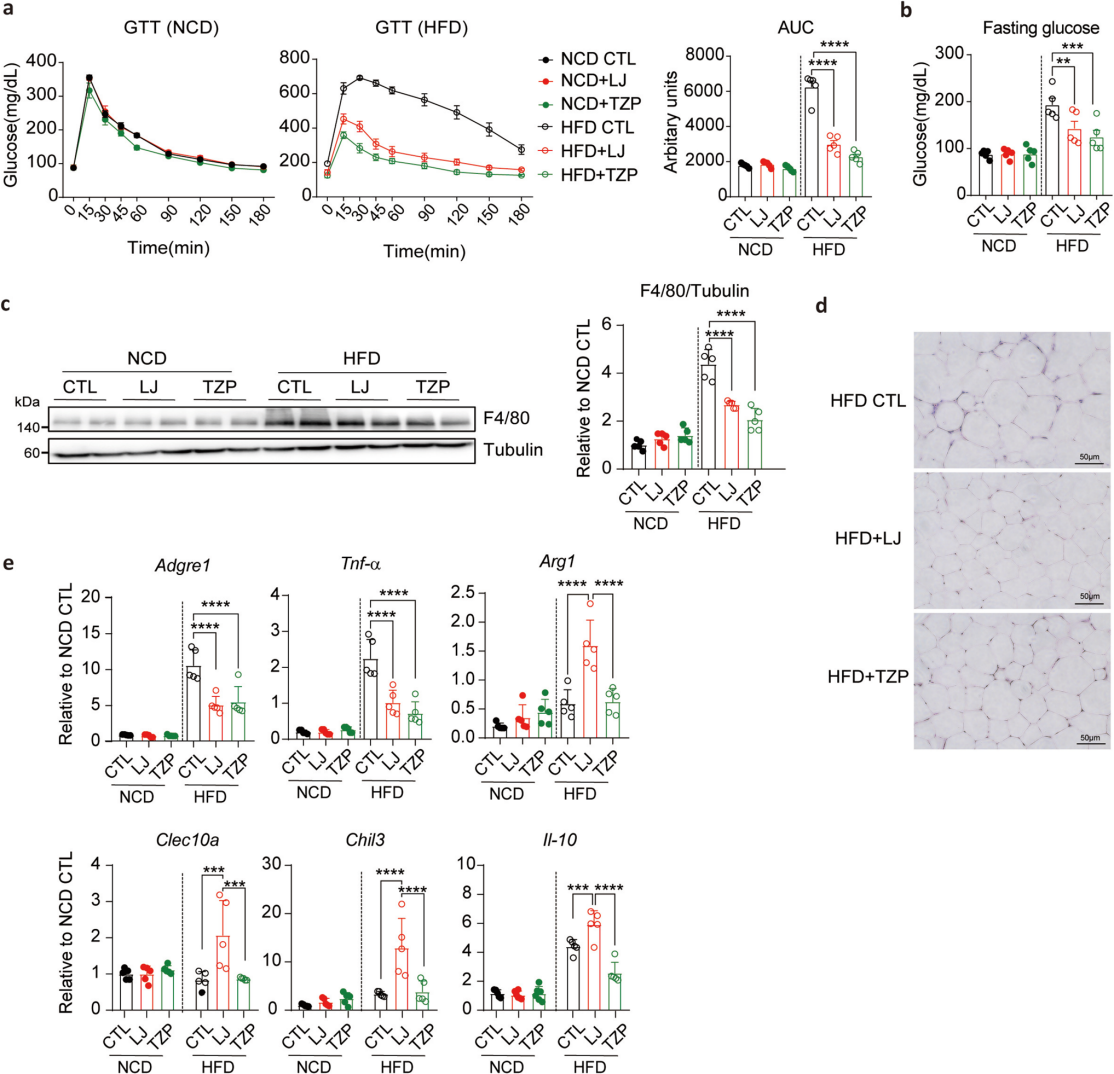
LJ4378 and TZP improve glucose intolerance and reduce inflammation in the gWAT of mice fed HFD.** a**. Intraperitoneal glucose tolerance test in NCD- or HFD-fed mice treated with vehicle, LJ-4378, or TZP. n = 5. Significant effects of diet (*p* < 0.0001) and treatment (*p* < 0.0001) were observed. **b**. Blood glucose level following a 12-h fast in NCD- or HFD-fed mice treated with vehicle, LJ-4378, or TZP. n = 5. Significant effects of diet (*p* = 0.0151) and treatment (*p* < 0.0001) were observed. **c**. Western blot analysis of F4/80, a marker of macrophage in gWAT of NCD- or HFD-fed mice treated with vehicle, LJ-4378, or TZP. n = 5. Significant effects of diet (*p* < 0.0001) and treatment (*p* < 0.0001) were observed. **d**. Hematoxylin and Eosin (H&E) staining analysis of gWAT in HFD-fed mice treated with vehicle, LJ-4378, or TZP. n = 5. Scale bar = 50 μm. **e**. RT-qPCR analysis of gene (*Adgre1*, *Tnf-α*, *Arg1, Clec10a, Chil3, Il-10*) expressions in gWAT of NCD- or HFD-fed mice treated with vehicle, LJ-4378, or TZP. n = 5. Significant effects of diet (*Adgre1*: *p* = 0.0001, *Tnf-α*: *p* < 0.0001, *Arg1*: *p* = 0.0001, *Clec10a*: *p* = 0.0056, *Chil3*: *p* = 0.0007, *Il-10*: *p* < 0.0001) and treatment (*Adgre1*: *p* < 0.0001, *Tnf-α*: *p* < 0.0001, *Arg1*: *p* < 0.0001, *Clec10a*: p = 0.1675, *Chil3*: p < 0.0001, *Il-10*: p < 0.0001) were observed. Statistical significance was determined using two-way ANOVA followed by Bonferroni post hoc test. Values are presented as mean ± SEM (^∗∗∗∗^*p* < 0.0001, ^∗∗∗^*p* < 0.001, ^∗∗^*p* < 0.01, ^∗^*p* < 0.05)

To assess the metabolic benefits of LJ-4378 and TZP, we examined obesity-induced metabolic dysfunction. Both treatments markedly enhanced glucose tolerance as indicated by an intraperitoneal glucose tolerance test (Fig. 2a). In addition, fasting glucose levels were significantly lower in the HFD-fed mice treated with LJ-4378 or TZP, indicating improved glycemic control (Fig. 2b).

Diet-induced obesity is characterized by chronic low-grade inflammation in adipose tissue, marked by increased macrophage infiltration in adipose tissue and the production of pro-inflammatory cytokines, which can be reduced by weight loss (Hildebrandt et al. 2023). Consistently, both LJ-4378 and TZP significantly reduced macrophage accumulation in gWAT, as indicated by decreased F4/80 protein expression and fewer crown-like structures (CLS) compared with HFD controls (Fig. 2c, d). This reduction was accompanied by a shift in gene expression towards an anti-inflammatory profile (Fig. 2e). Expression of adhesion G protein-coupled receptor E1 (*Adgre1*) and tumor necrosis factor-alpha (*Tnf-α*) was remarkably suppressed by both treatments, suggesting reduced pro-inflammatory signaling. Additionally, serum TNF-α levels were reduced in LJ-4378 and TZP-treated HFD-fed mice, indicating attenuation of both adipose tissue and systemic inflammation (Fig. S3). Notably, markers of M2-like macrophages, including arginase 1 (*Arg1*), C-type lectin domain containing 10A (*Clec10a*), chitinase-like 3 (*Chil3*), and interleukin 10 (*Il-10*), were selectively upregulated in the LJ-4378 group, indicating the promotion of anti-inflammatory macrophage polarization (Fig. 2e). Together, these data demonstrate that both LJ-4378 and TZP alleviate obesity-related metabolic dysfunctions, including glucose intolerance and gWAT inflammation.

### LJ-4378 and TZP treatment increase energy expenditure and enhance adipose tissue metabolism in HFD-fed mice

**Fig. 3:**
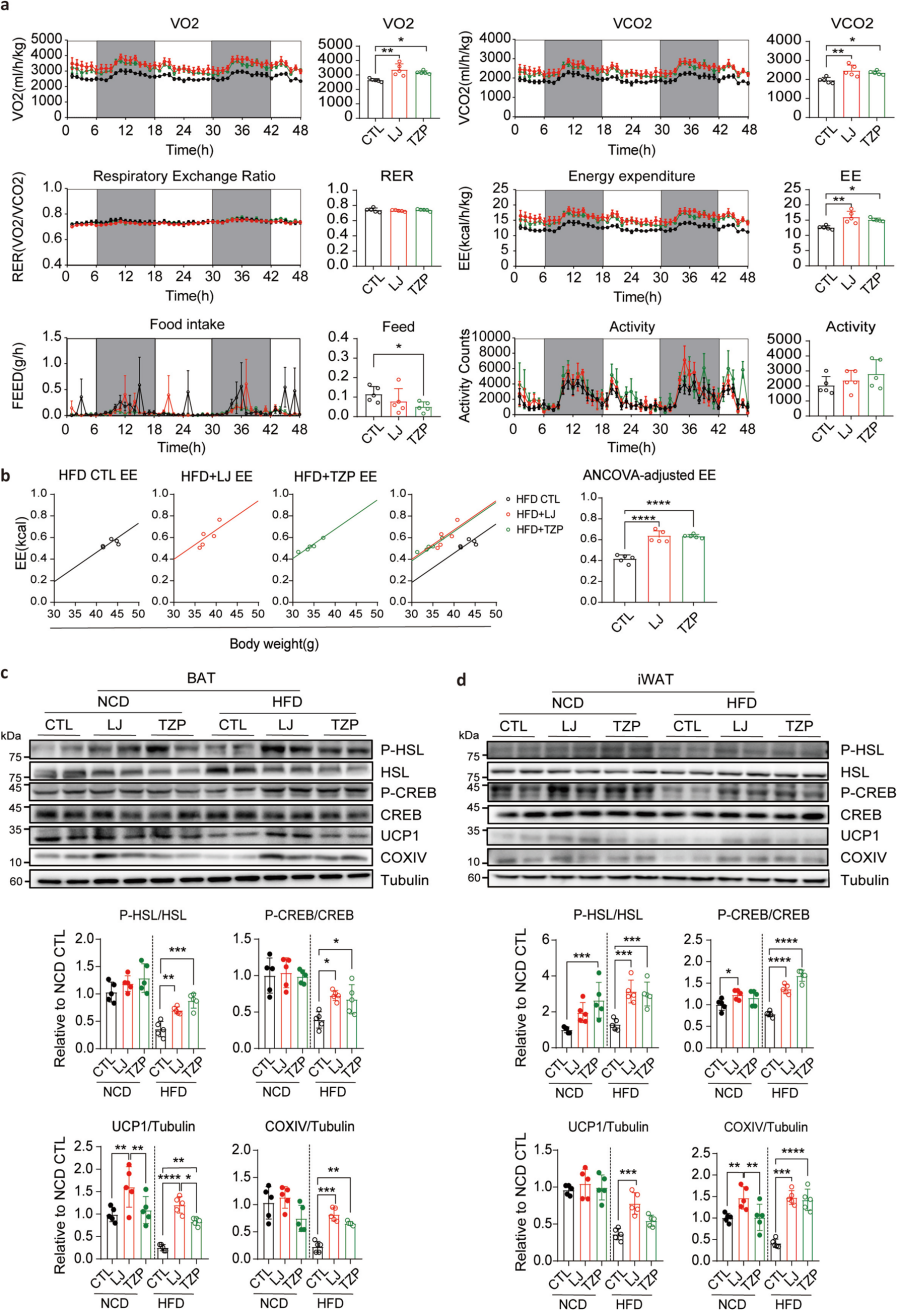
LJ-4378 and TZP enhance energy metabolism in HFD-fed mice. **a**. Indirect calorimetry analysis of HFD-fed mice treated with vehicle, LJ-4378, or TZP. n = 5. Significant group effects (volume of oxygen consumed (VO_2_): *p* = 0.0020, carbon dioxide produced (VCO_2_): *p* = 0.0046, respiratory exchange ratio (RER): *p* = 0.7300, Energy expenditure (EE): *p* = 0.0022, Feed: *p* = 0.0604, Activity: *p* = 0.3080) were observed. **b.** ANCOVA analysis of EE with body weight as a covariate in HFD-fed mice treated with vehicle, LJ-4378, or TZP. n = 5. **c**, **d**. Western blot analysis of phosphorylated hormone-sensitive lipase (P-HSL) and mitochondrial proteins (phosphorylated cAMP response element-binding protein (P-CREB), uncoupling protein 1 (UCP1), and cytochrome c oxidase subunit 4 (COXIV)) in BAT and iWAT of NCD- or HFD-fed mice treated with vehicle, LJ-4378, or TZP. n = 5. Significant effects of diet (P-HSL/HSL(BAT): *p* < 0.0001, P-CREB/CREB(BAT): *p* = 0.0523, UCP1/Tubulin(BAT): *p* < 0.0001, COXIV/Tubulin(BAT): *p* = 0.0009 and P-HSL/HSL(iWAT): *p* < 0.0001, P-CREB/CREB(iWAT): *p* < *0.0001*, UCP1/Tubulin(iWAT): *p* = 0.0012, COXIV/Tubulin(iWAT): *p* < 0.0001) and treatment (P-HSL/HSL(BAT): *p* < 0.0001, P-CREB/CREB(BAT): *p* < 0.0001, UCP1/Tubulin(BAT): *p* < 0.0001, COXIV/Tubulin(BAT): *p* < 0.0001 and P-HSL/HSL(iWAT): *p* = 0.0125, P-CREB/CREB(iWAT): *p* = 0.0048, UCP1/Tubulin(iWAT): *p* < 0.0001, COXIV/Tubulin(iWAT): *p* = 0.4516) were observed. Statistical significance was determined using one-way ANOVA followed by Bonferroni post hoc test in **a**, ANCOVA analysis in **b**, and two-way ANOVA followed by Bonferroni post hoc test in **c** and **d**. Values are presented as mean ± SEM (^∗∗∗∗^*p* < 0.0001, ^∗∗∗^*p* < 0.001, ^∗∗^*p* < 0.01, ^∗^*p* < 0.05)

Both LJ-4378 and TZP enhance cAMP signaling in adipocytes and modulate energy metabolism (Kim et al. 2022; Yu et al. 2025). To directly compare their metabolic effects, we performed indirect calorimetry on HFD-fed mice treated with each compound. Both treatments significantly increased oxygen consumption (VO_2_), carbon dioxide production (VCO_2_), and energy expenditure relative to HFD controls, indicating enhanced metabolic activity (Fig. 3a). After adjusting for body weight and lean body mass (LBM) using analysis of covariance (ANCOVA), only LJ-4378 consistently increased energy expenditure across the models, whereas TZP was not significant when LBM was used as the covariate (Fig. 3b and Fig. S4a). Interestingly, the two compounds exhibited distinct effects on food intake. TZP reduced food consumption, whereas LJ-4378 did not affect the feeding behavior (Fig. 3a). These findings suggest that, in line with previous reports (Geisler et al. 2023), TZP promotes weight loss at least partially by regulating appetite, whereas LJ-4378 promotes weight loss regardless of food intake.

To further elucidate the metabolic effects of LJ-4378 and TZP, we examined the expression of lipolytic and thermogenic regulators in the adipose tissue (Fig. 3c, d). In BAT, HFD-fed mice treated with either compound led to elevated levels of phosphorylated hormone-sensitive lipase (P-HSL), phosphorylated cAMP response element-binding protein (P-CREB), uncoupling protein 1 (UCP1), and cytochrome c oxidase subunit 4 (COXIV) (Fig. 3c). A similar pattern of upregulation was observed in iWAT, suggesting that both LJ-4378 and TZP stimulated adipose tissue metabolism under obese conditions (Fig. 3d). Notably, UCP1 expression in BAT was more strongly induced by LJ-4378 than TZP, suggesting a more pronounced thermogenic response (Fig. 3d). This effect was likely attributable to the direct activation of PKA signaling by LJ-4378 (Kim et al. 2022).

Consistent with these findings, the proteins involved in mitochondrial oxidative phosphorylation were upregulated in HFD-fed mice treated with either compound alone (Fig. S5a, b). Despite increased lipolysis, serum free fatty acid (FFA) levels remained unchanged, consistent with enhanced mitochondrial oxidative phosphorylation, suggesting the efficient utilization of lipolysis-derived substrates for energy metabolism. (Fig. S5c).

To evaluate whether the observed effects extended beyond adipose tissue, we examined hepatic PKA signaling. Neither LJ-4378 nor TZP altered the phosphorylation of the downstream targets of PKA in the liver (Fig. S6). The metabolic improvement observed with treatment is likely attributable to its anti-obesity effects and consequent improvement in adipose tissue insulin sensitivity (Lee et al. 2023). However, the direct effects of LJ-4378 on hepatic lipid metabolism require further investigation.

### LJ-4378 maintains reduction in body weight and adiposity after treatment cessation compared to TZP

**Fig. 4:**
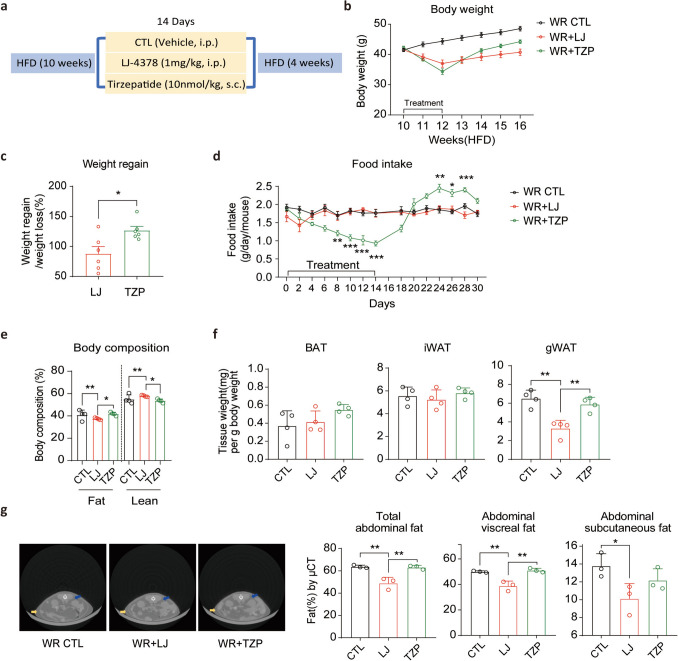
LJ-4378-treated mice exhibit lower weight regain than TZP-treated mice after treatment withdrawal. **a**. Schematic of the experimental design in the weight rebound (WR) model. Following 14 days of vehicle, LJ-4378, or TZP treatment in mice with diet-induced obesity, they underwent a 4-week withdrawal period with HFD feeding to induce weight regain. **b**. Body weight changes during the 14-day treatment and the 4-week post-treatment period in WR groups. n = 6. **c**. Percentage of body weight regained over 4 weeks in WR groups, relative to initial weight loss. n = 6. **d**. Food intake measurement during treatment and for 16 days of the post-treatment phase. n = 4. Significant group effects (Day 8: *p* = 0.0031, Day 10: *p* < 0.0001, Day 12: *p* = 0.0002, Day 14: *p* < 0.0001, Day 24: *p* = 0.0014, Day 26: *p* = 0.0104, Day 28: *p* = 0.0005) were observed. **e**. Percentage of body fat and lean mass in 4 weeks after treatment cessation in WR groups. n = 4. Significant group effects (Fat: *p* = 0.0064, Lean: *p* = 0.0033) were observed. **f.** Adipose tissue (BAT, iWAT, and gWAT) weights at 4 weeks after treatment cessation in WR groups. n = 4. Significant group effects (BAT: *p* = 0.3293, iWAT: *p* = 0.5368, gWAT: *p* = 0.0010) were observed. **g**. Representative micro-CT images and quantification of abdominal fat volumes (total, subcutaneous, and visceral) at 4 weeks post-treatment in WR groups. n = 3. (Yellow arrows indicate subcutaneous adipose depot, and blue arrows indicate visceral adipose depot) Significant group effects (Total abdominal fat: *p* = 0.0023, Abdominal visceral fat: *p* = 0.0014, Abdominal subcutaneous fat: *p* = 0.0568) were observed. Statistical significance was determined using an unpaired two-tailed *t*-test in **c** and one-way ANOVA followed by Bonferroni post hoc test in **d–g**. Values are presented as mean ± SEM (^∗∗∗^*p* < 0.001, ^∗∗^*p* < 0.01, ^∗^*p* < 0.05)

A major challenge in the development of anti-obesity drugs is achieving durable weight loss and long-term metabolic improvement (van Baak and Mariman. 2025). To investigate the potential for weight regain after treatment discontinuation, we used an in vivo weight rebound (WR) model (Fig. 4a). Body weight was monitored throughout the treatment and 4 weeks post-cessation (Fig. 4b). Mice previously treated with TZP exhibited rapid weight regain, returning to baseline within 2 weeks and surpassing the initial body weight by 4 weeks. In contrast, the LJ-4378 mice maintained a relatively low body weight post-treatment. (Fig. 4b). When expressed as a percentage of the initial weight loss, LJ-4378-treated mice recovered 88% of their body mass, whereas TZP-treated mice exhibited a 127% loss (Fig. 4c).

To explore whether energy intake contributed to the observed weight regain, we monitored food consumption during the 14-day treatment and period and 16 days after cessation (Fig. 4d). During administration, TZP significantly suppressed food intake. However, upon withdrawal, the mice exhibited a sharp rebound in food consumption. By day 6 post-treatment, food intake in the TZP group exceeded that in both the WR controls and the LJ-4378 group. This post-treatment hyperphagia closely paralleled the body weight regain, suggesting that rebound feeding contributed substantially to the transient efficacy of TZP. In contrast, LJ-4378 did not alter food intake during or after treatment, which was consistent with its sustained effect on weight reduction.

The assessment of adiposity after treatment cessation revealed a more favorable profile in the LJ-4378 group than in the TZP group. The mice treated with LJ-4378 exhibited a significantly lower whole-body fat ratio than in those treated with TZP (Fig. 4e). Volumetric analysis of adipose depots revealed a reduction in gWAT and visceral abdominal fat in the LJ-4378-treated group, compared with the TZP group (Fig. 4f, g). These findings suggest that LJ-4378 not only limits post-treatment weight regain but also supports long-term reductions in adiposity.

### LJ-4378 maintains metabolic improvements more effectively than TZP in a weight rebound model

**Fig. 5:**
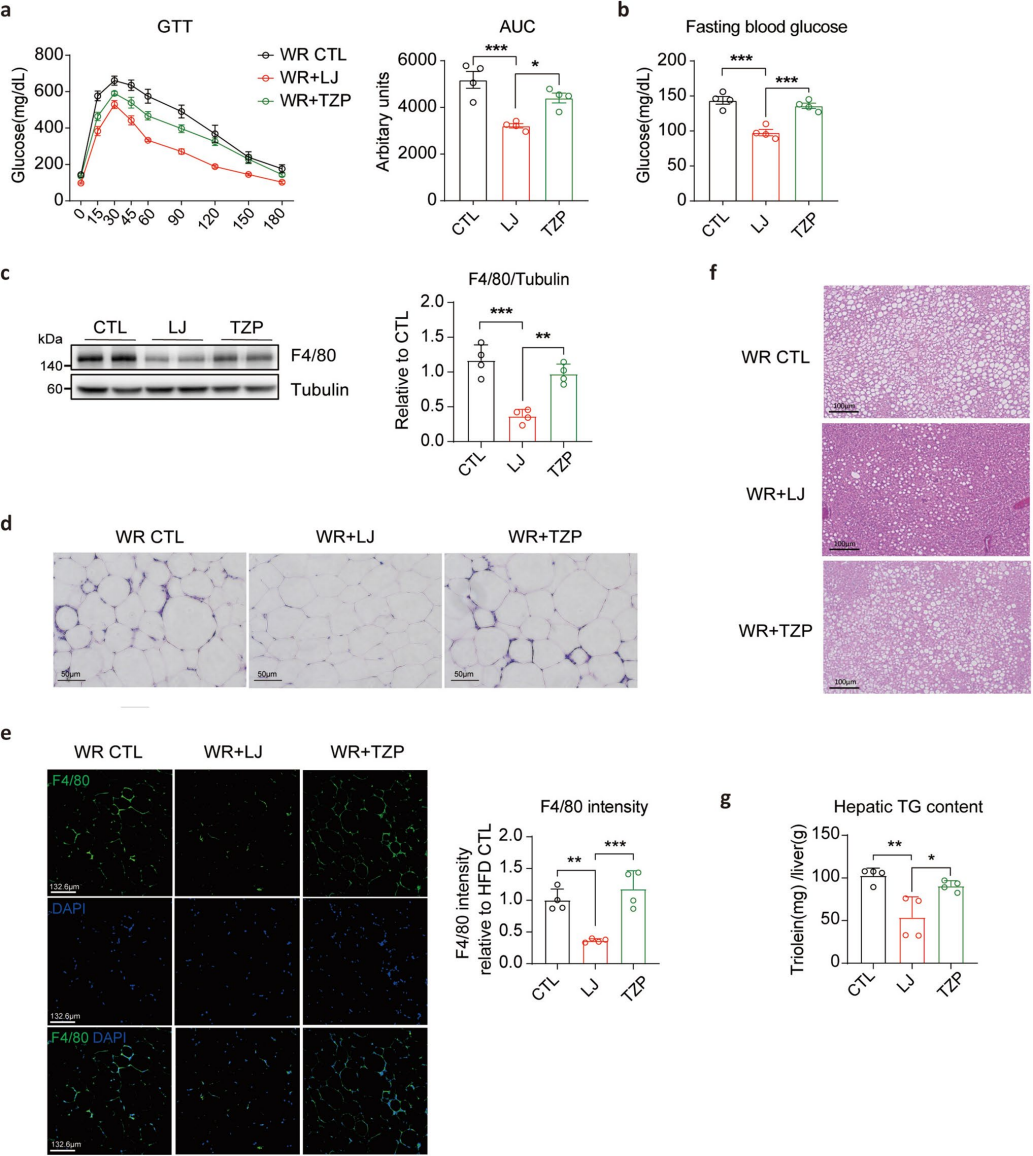
Following treatment withdrawal, LJ-4378 better preserves metabolic improvements in obesity-related dysfunctions compared to TZP. **a**. Intraperitoneal glucose tolerance test at 4 weeks post-treatment in WR groups. n = 4. Significant group effects (*p* = 0.0011) were observed. **b.** Blood glucose levels following a 12-h fast at 4 weeks post-treatment in WR groups. n = 4. Significant group effects (*p* = 0.0001) were observed. **c**. Western blot analysis of F4/80, a marker of macrophage, at 4 weeks of post-treatment cessation in WR groups. n = 4. Significant group effects (*p* = 0.0002) were observed. **d**. H&E staining analysis of gWAT at 4 weeks post-treatment in WR groups. n = 4. Scale bar = 50 μm. **e**. Immunohistochemistry analysis and quantification of F4/80, the marker of macrophage, in gWAT at 4 weeks post-treatment in WR groups. n = 4. Scale bar = 132.6 μm. Significant group effects (*p* = 0.0006) were observed. **f**. H&E staining of the liver at 4 weeks post-treatment in WR groups. n = 4. Scale bar = 100 μm. **g**. Quantification of TG accumulation in the liver at 4 weeks post-treatment in WR groups. n = 4. Significant group effects (*p* = 0.0037) were observed. Statistical significance was determined using one-way ANOVA followed by Bonferroni post hoc test. Values are presented as mean ± SEM (^∗∗∗∗^*p* < 0.0001, ^∗∗∗^*p* < 0.001, ^∗∗^*p* < 0.01, ^∗^*p* < 0.05)

To evaluate the durability of the metabolic benefits following treatment cessation, we analyzed glucose metabolism, adipose tissue inflammation, and hepatic steatosis at 4 weeks after the WR period. In the glucose tolerance test, mice treated with LJ-4378 displayed enhanced glucose clearance and lower fasting glucose levels than those in the TZP-treated group, indicating sustained glycemic improvement (Fig. 5a, b).

Weight regain following dietary intervention is associated with macrophage infiltration and inflammatory responses in the visceral adipose tissue (Sougiannis et al. 2020). To investigate adipose tissue inflammation in the WR model, we assessed macrophage infiltration in the gWAT. In the TZP-treated group, the expression of the macrophage marker F4/80 (encoded by *Adgre1*) and the formation of CLS, which had been reduced by treatment, were re-elevated following treatment cessation (Fig. 5c–e, Fig. S7). In contrast, the LJ-4378-treated group maintained lower levels of macrophage accumulation after 4 weeks of the weight regaining phase (Fig. 5c–e, Fig. S7). Furthermore, reduced *Tnf-α* and increased *Arg1* mRNA levels were maintained after 4 weeks of WR (Fig. S7).

Additionally, weight cycling in mice with a history of chronic diet-induced obesity leads to increased hepatic lipid storage despite weight loss compared to those maintained on a consistent diet (Bernecker et al. 2025). In our WR model, histological and biochemical analyses revealed significantly lower hepatic triglyceride (TG) deposition in the LJ-4378-treated group than in the TZP (Fig. 5f, g). Notably, the TZP-treated group, which experienced greater weight regain, also showed a resurgence of obesity-associated metabolic impairments compared with the LJ-4378 administration. These findings underscore the superior long-term metabolic protection conferred by LJ-4378 after treatment cessation.

### LJ-4378-treated mice sustain enhanced energy metabolism compared with TZP-treated mice after treatment discontinuation

**Fig. 6:**
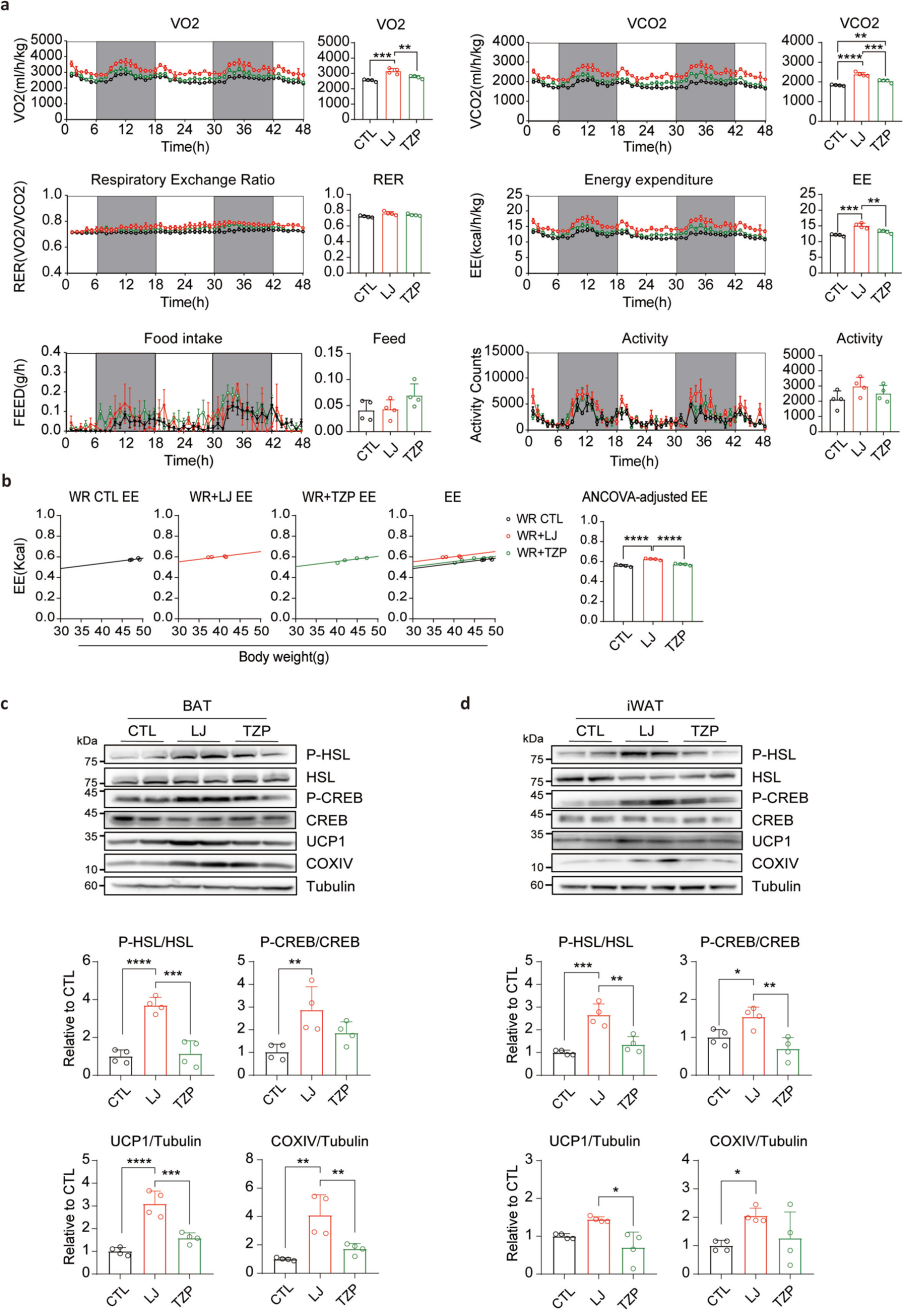
LJ-4378-treated mice sustain enhanced energy metabolism compared to TZP-treated mice after treatment discontinuation. **a** Indirect calorimetry analysis at 2 weeks post-treatment in WR groups. n = 4. Significant groups effects (VO_2_: *p* = 0.0003, VCO_2_: *p* < 0.0001, RER: *p* = 0.0273, EE: *p* = 0.0002, Feed: *p* = 0.0874, Activity: *p* = 0.1501) were observed. **b** ANCOVA analysis of EE with body weight as a covariate at 2 weeks post-treatment in WR groups. n = 4. **c**, **d**. Western blot analysis of P-HSL and mitochondria proteins (P-CREB, UCP1, and COXIV) in BAT and iWAT at 2 weeks post-treatment in WR groups. n = 4. Significant group effects (P-HSL/HSL(BAT): *p* < 0.0001, P-CREB/CREB(BAT): *p* = 0.0124, UCP1/Tubulin(BAT): *p* < 0.0001, COXIV/Tubulin(BAT): *p* = 0.0016 and P-HSL/HSL(iWAT): *p* = 0.0002, P-CREB/CREB(iWAT): *p* = 0.0038, UCP1/Tubulin(iWAT): *p* = 0.0061, COXIV/Tubulin(iWAT): *p* = 0.0461) were observed. Statistical significance was determined using one-way ANOVA followed by Bonferroni post hoc test. in **a**, **c**, and **d**, and ANCOVA analysis in **b**. Values are presented as mean ± SEM (^∗∗∗∗^*p* < 0.0001, ^∗∗∗^*p* < 0.001, ^∗∗^*p* < 0.01, ^∗^*p* < 0.05)

Weight regain following the discontinuation of anti-obesity therapy is often linked to a reduction in resting energy expenditure (Bosy-Westphal et al. 2013; Christoffersen et al. 2022). To investigate this relationship, we assessed the energy metabolism in the WR model using indirect calorimetry (Fig. 6a). Two weeks after treatment cessation, LJ-4378-treated mice demonstrated elevated VO_2_, VCO_2_, and energy expenditure compared with those in WR controls. However, these metabolic parameters were significantly higher in the LJ-4378 than in the TZP-treated group, indicating a more sustained enhancement of energy metabolism following treatment cessation (Fig. 6a). Consistently ANCOVA, adjusted for body mass, showed that the LJ-4378 group had higher energy expenditure than both groups treated with vehicle or TZP (Fig. 6b). Furthermore, after adjusting for LBM, LJ-4378 exhibited higher energy expenditure to the WR + TZP (Fig. S4b). After 4 weeks of WR, the elevated energy expenditure in the LJ-4378-treated group was no longer evident and returned to levels comparable to those in the control group (Fig. S8).

The transient hyperphagia observed immediately after TZP withdrawal (Fig. 4d) appeared to be a short-term effect; however, it likely contributed to accelerated weight regain.

To evaluate the metabolic function of adipose tissue at the molecular level, we examined the expression of P-HSL and mitochondrial proteins in BAT and iWAT 2 weeks after treatment cessation (Fig. 6c, d). Mice previously treated with LJ-4378 expressed higher levels of P-HSL and mitochondrial proteins than those in both WR controls and the TZP-treated group, indicating sustained activation of lipolysis and mitochondrial function in BAT and iWAT. These findings suggest that LJ-4378 promotes prolonged adipose tissue metabolic activity compared with TZP, contributing to its capacity to maintain energy homeostasis and prevent post-treatment metabolic decline.

### Evaluation of pharmacokinetic and tissue distribution properties of LJ-4378

To assess the drug properties and therapeutic potential of LJ-4378, we comprehensively evaluated its pharmacokinetic characteristics using in vitro and in vivo models. Plasma protein binding assays showed free fractions of 30.1% (human), 29.7% (rat), and 12.7% (mouse), which were within the acceptable range for orally administered small-molecule drugs (Table 3). Plasma stability studies demonstrated that LJ-4378 remained highly stable for over 120 min, with more than 90% of the compound retained in human, rat, and mouse plasma (Table 4).

**Table 3:** Plasma free fraction (% Free) of LJ-4378 in Human, Rat, and Mouse (Plasma Protein Binding (PPB) assay)

% Free Compound	Human	Rat	Mouse
LJ-4378	30.1	29.7	12.7
Dexamethasone (Reference)	35.9	22.2	29.7
Warfarin (Reference)	0.6	1.0	6.2

**Table 4:** Plasma Stability of LJ-4378 in Human, Rat, and Mouse

% Remaining compound	Human		Rat		Mouse	
	30 min	120 min	30 min	120 min	30 min	120 min
LJ-4378	97.0	94.4	> 100	95.2	98.4	91.2
Procaine (Reference)	1.2 (5 min)	< 1 (10 min)	85.2	45.2	7.0	< 1
Enalapril (Reference)	> 100	95.9	34.5	1.4	66.0	26.3

In vivo pharmacokinetic analysis in mice revealed dose-dependent exposure following I.V. and P.O. (Table 5). After a single intravenous dose of 2 mg/kg, LJ-4378 showed C_max_ of 2395.23 ng/mL, half-life of 0.46 h, and systemic clearance of 80.7 mL/min/kg. Oral administration resulted in dose-dependent pharmacokinetics, with bioavailability increasing from 62.93% at 10 mg/kg to 86.08% at 50 mg/kg.

**Table 5:** Pharmacokinetic parameters of LJ-4378 in mice following single intravenous and oral administration

PK parameter	Route of dosing			
	I.V. (n = 3)	P.O. (n = 3)	P.O. (n = 3)	P.O. (n = 3)
Dose (mg/kg)	2.00	10.00	20.00	50.00
AUC_0-t_ (h·ng/mL)	411.45	1273.67	3067.21	8446.78
C_max_ (ng/mL)	2395.23	1220.57	2401.74	5307.60
T_max_ (h)	0.03	0.08	0.25	0.50
CL ((mL/min)/kg)	80.7	128.5	103.2	93.0
t_1/2_ (h)	0.46	2.64	3.89	3.11
F (%)	–	62.93	78.42	86.08

Tissue distribution analysis in SD rats 240 min after intravenous infusion showed that plasma concentrations reached a steady state by 240 min and remained constant thereafter (*p* = 0.525) (Fig. S9). The steady-state tissue-to-plasma partition coefficients ($K_{P,T}$) were 0.440, 0.228, 0.0843, and 0.00187 for the liver, lungs, adipose tissue, and brain, respectively (Table 6). Notably, the adipose tissue exhibited over four-fold higher partitioning than that in the brain, indicating a preferential distribution to the peripheral tissues. Consistent with its tissue distribution profile, LJ-4378 did not alter hypothalamic Fos proto-oncogene (*Fos*), pro-opiomelanocortin-alpha (*Pomc*), and neuropeptide Y (*Npy*) expression (Fig. S10), indicating negligible central penetration.

**Table 6:** ${\varvec{K}}_{{{\varvec{P}},{\varvec{T}}}}$ for LJ-4378 in four representative tissues in SD rats. The rat ${\varvec{K}}_{{{\varvec{P}},{\varvec{T}}}}$ values were also extrapolated to those in mice and humans under the assumption of free drug hypothesis and consistent tissue unbound fraction

Steady state tissue partition coefficient ($K_{P,T}$)			
	SD rats (observed)	Mice (extrapolated)	Humans (extrapolated)
Adipose	0.0843 ± 0.00653	0.197	0.200
Brain	0.0187 ± 0.00419	0.0437	0.0443
Liver	0.440 ± 0.194	1.03	1.04
Lungs	0.228 ± 0.0793	0.533	0.540

Extrapolation of these $K_{P,T}$ values to mice and humans, using species-specific plasma unbound fractions and assuming the free drug hypothesis with consistent tissue unbound fractions, suggests that this distribution trend is likely to be maintained across species. Collectively, these characteristics indicate that LJ-4378 achieves sufficient adipose tissue exposure to mediate its pharmacological effects, while displaying favorable drug-like properties and translational potential for further development.

## Discussion

In this study, we directly compared the anti-obesity efficacy of LJ-4378, a dual A_2A_ agonist/A_3_ antagonist, with that of TZP, a dual GLP-1/GIP receptor agonist with proven clinical efficacy. We aimed to assess not only the acute effects of these agents on weight loss and metabolic health but also their impact on weight regain after treatment cessation. Using a diet-induced obesity model and weight rebound paradigm, we found that both compounds reduced body weight and adiposity, improved glucose tolerance, and alleviated adipose tissue inflammation. However, the mechanisms underlying these effects are distinct, and only LJ-4378 attenuated weight regain by sustaining energy metabolism and preventing compensatory hyperphagia. These findings suggest that targeting the adipose tissue energy metabolism may represent a complementary therapeutic strategy for the regulation of incretin-based appetite in obesity management.

Mechanistically, LJ-4378 acts through dual modulation of adenosine receptors. A_2A_ receptor activation stimulates adenylyl cyclase via Gs signaling, whereas A_3_ receptor antagonism prevents Gi-mediated inhibition, augments intracellular cAMP, and activates the PKA pathway (Im et al. 2021). This cascade enhances lipolysis, mitochondrial function, and thermogenic activation in adipocytes, leading to improved systemic energy expenditure and adipose tissue remodeling. Consistent with these mechanisms, LJ-4378 elevates cAMP more strongly than selective A_2A_ or A_3_ ligands and drives adipose browning and weight reduction in preclinical models (Kim et al. 2022). In contrast, TZP acts primarily via incretin signaling. GLP-1 receptor activation enhances glucose-dependent insulin secretion and suppresses appetite, whereas GIP receptor activation improves insulin sensitivity and regulates lipid metabolism and thermogenesis in the adipose tissue (Regmi et al. 2024; Yu et al. 2025). Thus, the efficacy of TZP is derived from both central appetite suppression and peripheral metabolic improvements, whereas LJ-4378 primarily targets adipose tissue lipid metabolism through adenosine receptor pathways that promote lipolysis and thermogenesis independent of GIP signaling. Accordingly, the present side-by-side comparison highlights their distinct therapeutic strategies and suggests that LJ-4378 may provide less potent but more durable efficacy, with a lower risk of weight regain, while offering a complementary approach to incretin-based therapy. Given these distinct yet potentially complementary mechanisms, the co-administration of LJ-4378 with existing GLP-1 receptor agonists may offer additive benefits for weight reduction and metabolic regulation, although this remains hypothetical and requires validation in dedicated studies.

TZP, used as a comparator in this study, is the most potent anti-obesity drug, superior to other classes of anti-obesity drugs. It has consistently demonstrated robust weight loss, improved glycemic control, and favorable cardiometabolic outcomes, while also exhibiting good safety and tolerability in clinical studies (Jastreboff et al. 2022; Sattar et al. 2022). However, in our in vivo WR model, the glycemic improvement induced by TZP was lost after treatment withdrawal, whereas clinical studies have reported that patients often maintain glycemic benefits despite partial weight regain (Gasoyan et al. 2025). One important distinction is that our WR groups were maintained on a 60% HFD throughout the rebound phase, a condition not directly comparable to that of patients on long-term TZP treatment, who typically follow more varied or diet-controlled regimens for weight maintenance. In our study, TZP treatment was associated with a sustained reduction in food intake, whereas previous studies have described variable outcomes, including transient or partially persistent appetite suppression (Geisler et al. 2023; Liang et al. 2025; Ravussin et al. 2025). The difference between our experimental findings and those of previous reports likely reflects interspecies physiology, study duration, and diet composition, underscoring the need for further investigation into the long-term durability of anti-obesity drug effects.

Current anti-obesity therapies face several challenges, including weight regain and reduced resting energy expenditure (Bosy-Westphal et al. 2013; Christoffersen et al. 2022; Wilding et al. 2022). Our comparison revealed that while TZP produced greater weight loss, this effect was reversed upon treatment cessation, leading to rebound hyperphagia and weight regain. In contrast, LJ-4378 attenuated weight regain during the withdrawal phase without significantly altering food intake. Moreover, the difference in WR mice treated with LJ-4378 and TZP was attributed to their distinct effects on energy expenditure. Both agents enhanced whole-body energy expenditure during treatment; however, only LJ-4378 sustained elevated expenditure during the WR period. These findings suggest that the metabolic benefits of LJ-4378, likely due to its direct action on the adipose tissue, may provide a more durable basis for long-term weight control than TZP’s dependence on central appetite suppression. However, as this effect was not fully maintained throughout the extended 4-week withdrawal period, further evaluation in additional models beyond our continuous 60% HFD challenge is necessary to fully assess the metabolic benefits of LJ-4378.

LJ-4378 and TZP elicited distinct immunometabolic responses in gWAT. Both treatments reduced macrophage infiltration and pro-inflammatory cytokine levels in gWAT, which is consistent with their systemic metabolic benefits. LJ-4378 uniquely upregulates M2-like macrophage markers, including *Arg1*, *Clec10a*, *Chil3*, and *Il-10*, suggesting a shift toward anti-inflammatory macrophage polarization, whereas TZP does not alter M2-like macrophage genes (Xia et al. 2024). Immune remodeling, which has been implicated in the regulation of metabolic responses during weight cycling, may also play a critical role in sustaining metabolic improvements following drug treatment (Cottam et al. 2022). LJ-4378’s ability to modulate adipose tissue inflammation beyond weight reduction may be an additional therapeutic advantage. Further investigation of other immune cell populations is necessary to fully understand the broader immunometabolic effects on weight regain.

Given that obesity therapy often requires chronic administration, the safety and pharmacokinetic properties of candidate compounds are critical determinants of their clinical potential. LJ-4378 demonstrated a favorable preclinical safety profile, with no evidence of major toxicities or liabilities commonly associated with adenosine receptor modulators, which is consistent with previous reports on A_2A_/A_3_-targeting compounds (Brink et al. 2015; Schmidt and Ferk. 2017). In addition, LJ-4378 exhibited pharmacokinetic characteristics that supported its therapeutic development, including oral bioavailability, dose-proportional exposure, and preferential distribution in adipose tissue while showing negligible brain penetration. These features suggest a primarily peripheral mechanism of action that could be advantageous for the treatment of obesity. Taken together, the safety and pharmacokinetic attributes of LJ-4378 provide a strong rationale for its further evaluation as a potential long-term therapy for obesity.

By comparing LJ-4378 and TZP, our study provides evidence supporting the efficacy of LJ-4378 as a potential anti-obesity therapy. Our findings demonstrate that LJ-4378 promotes weight loss and metabolic health by enhancing energy metabolism in the adipose tissue, independent of appetite regulation. Notably, LJ-4378 maintained its metabolic benefits even after treatment cessation, likely due to its persistent effects on adipose tissue thermogenesis and substrate utilization. These findings underscore the advantage of targeting energy metabolism directly, without altering appetite, to achieve more durable anti-obesity outcomes and highlight LJ-4378’s potential as a sustainable metabolic intervention.

## Supplementary Information

Below is the link to the electronic supplementary material.

Supplementary file1 (DOCX 3072 KB)

## Data Availability

The datasets used and/or analyzed during the current study are available from the corresponding authors on reasonable request.
