# Supplementary material for: Comparative analysis of LJ-4378 and tirzepatide in mouse models of obesity and weight regain
Source: Arch Pharm Res. 2025 Nov 7;48(11-12):1441–59. doi: 10.1007/s12272-025-01575-9 (PMC12680861; doi:10.1007/s12272-025-01575-9)
Supplement: Supplementary file 1 — Supplementary file1 (DOCX 3072 KB) [file 12272_2025_1575_MOESM1_ESM.docx]

**Table S1. CYP Inhibition Profile of LJ-4378**

| Compound | % of control activity (at 10 µM) | | | | |
| --- | --- | --- | --- | --- | --- |
|  | CYP1A2 | CYP2C9 | CYP2C19 | CYP2D6 | CYP3A4 |
| LJ-4378 | 82.2 | 95.8 | 85.3 | 89.3 | 96.9 |
| Ketoconazole  (Reference) | 98.3 | 98.0 | > 100 | > 100 | 28.9 |


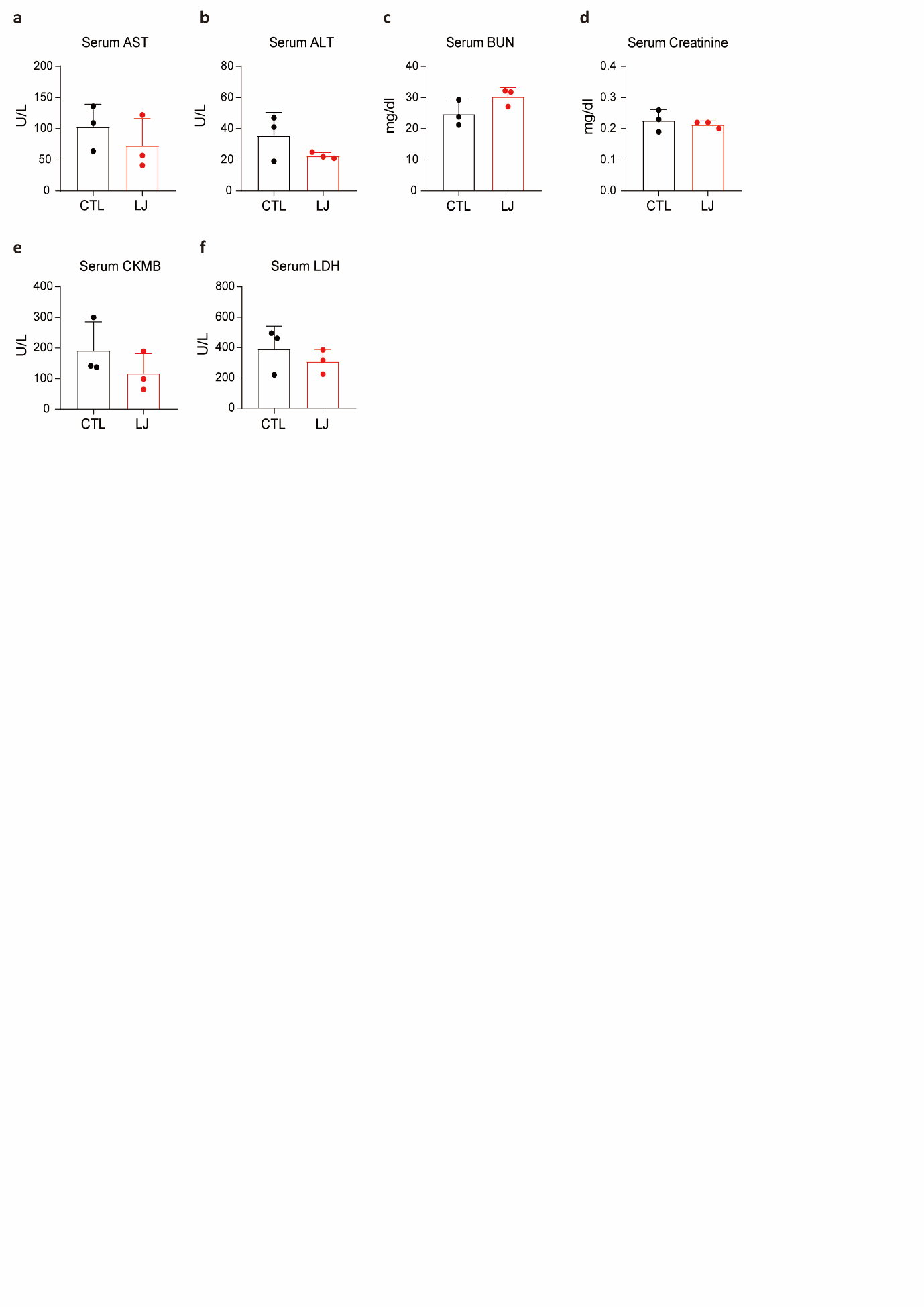


**Fig. S1 Effect of 2-week LJ-4378 treatment on serum markers of hepatic, renal, and cardiac toxicity in mice**

**a-f.** Serum levels of aspartate transaminase (AST), alanine transaminase (ALT), blood urea nitrogen (BUN), creatinine, creatine kinase MB (CKMB), and lactate dehydrogenase (LDH) in 2 weeks of LJ-4378 (1mg/kg) treatment in mice. n = 3. Statistical significance was determined using an unpaired two-tailed *t*-test.


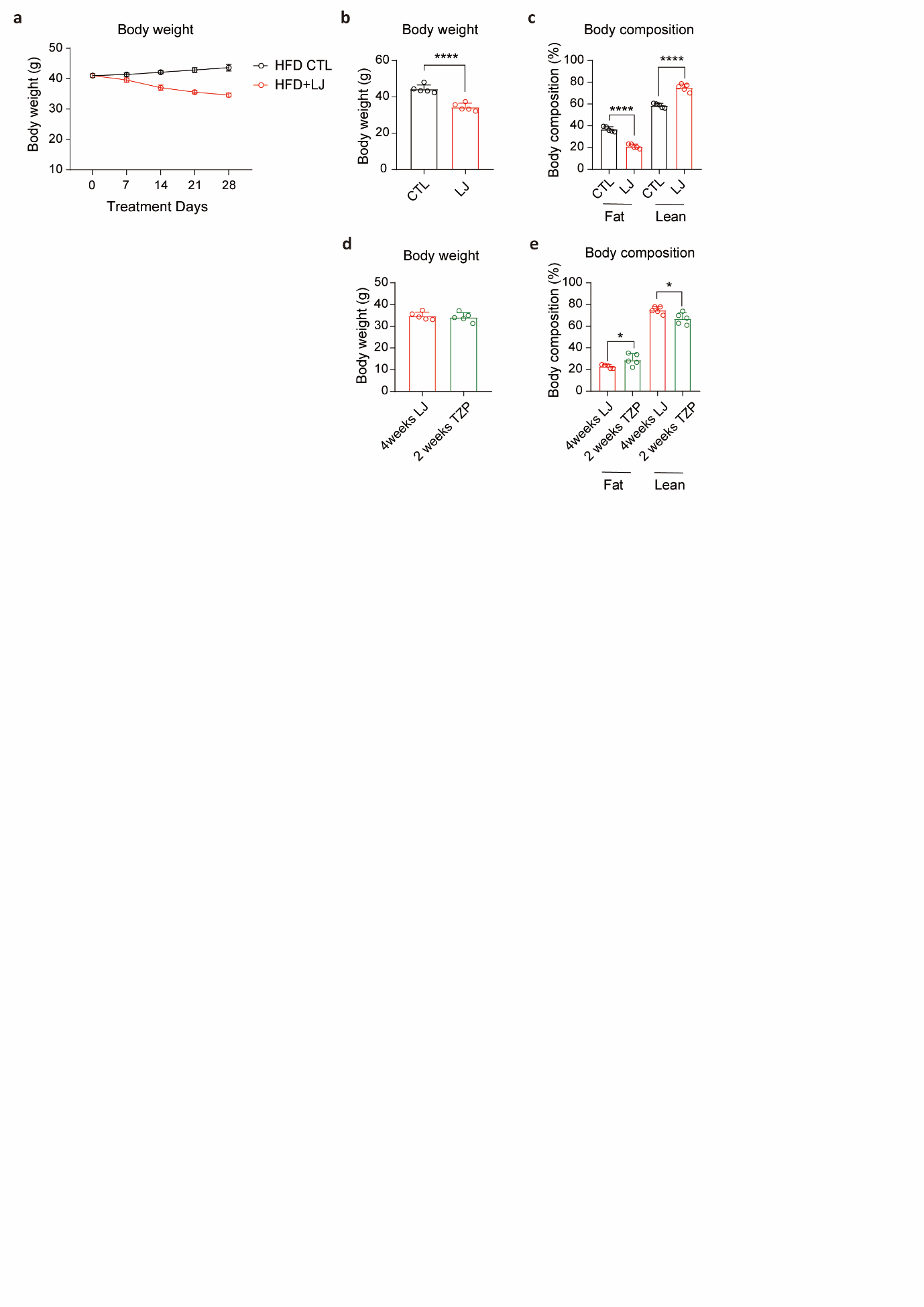


**Fig. S2. Effects of 4 weeks of LJ-4378 treatment on body weight loss and fat mass in HFD-fed mice.**

**a.** Body weight monitoring during 4 weeks of treatment with vehicle or LJ-4378. n = 5. **b.** Body weight after 4 weeks of treatment with vehicle or LJ-4378. n = 5. **c.** Percentage of whole-body fat and lean ratio after 4 weeks of treatment with vehicle or LJ-4378. n = 5. **d.** Body weight comparison between 4 weeks of LJ-4378 and 2 weeks of TZP treatment. n = 5. **e.** Body composition comparison between 4 weeks of LJ-4378 and 2 weeks of TZP treatment. n = 5. Statistical significance was determined using an unpaired two-tailed *t*-test. Values are presented as mean ± SEM (^∗∗∗^*p* < 0.001, ^∗∗^*p* < 0.01).


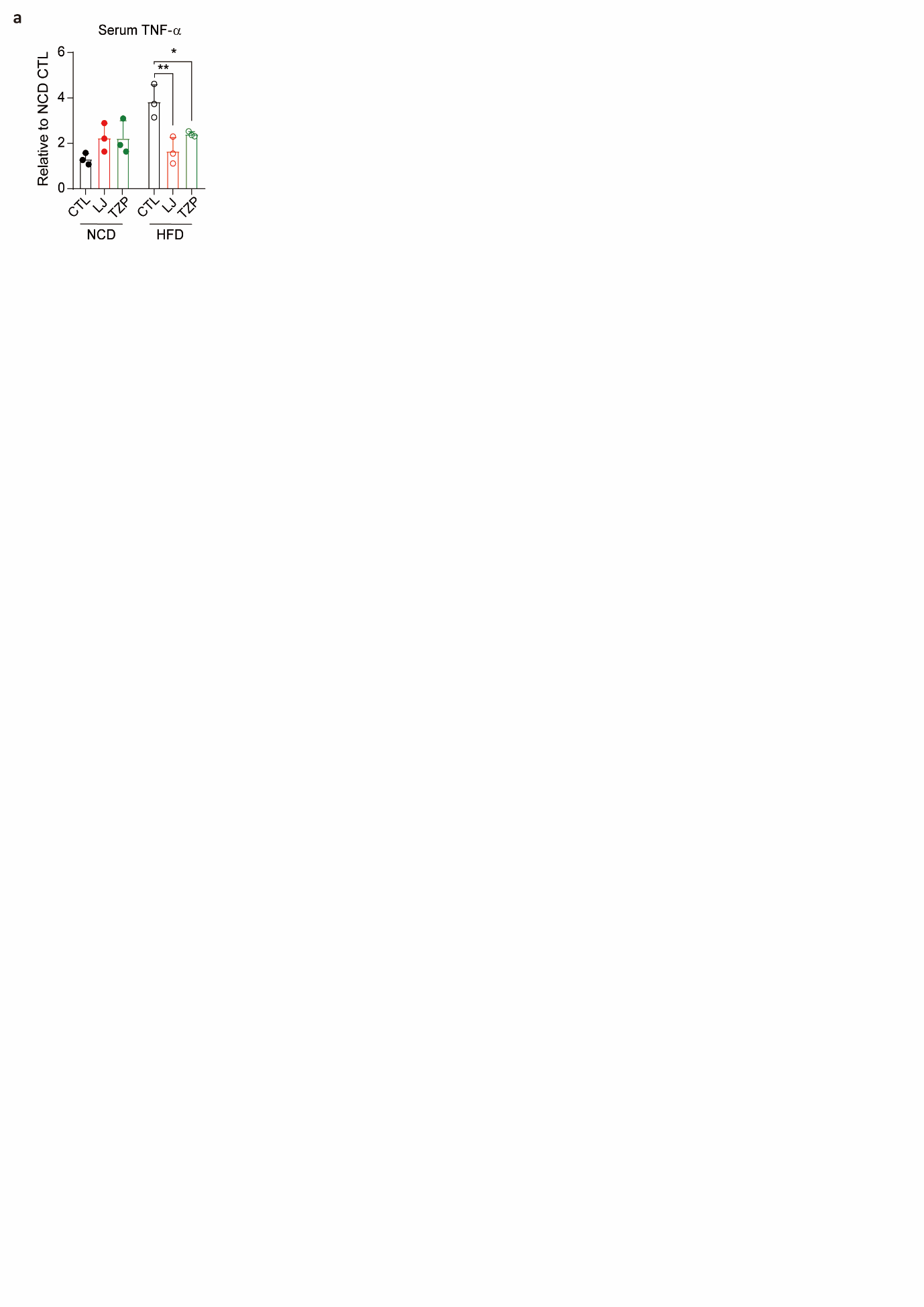


**Fig. S3. LJ-4378 and TZP treatment reduce serum TNF-α levels in HFD-fed mice.**

Serum levels of TNF-α in NCD- or HFD-fed mice treated with vehicle, LJ-4378, or TZP. n = 3. Significant effects of treatment (*p* = 0.0120) were observed. Statistical significance was determined using two-way ANOVA followed by Bonferroni post hoc test. Values are presented as mean ± SEM (^∗∗^*p* < 0.01, ^∗^*p* < 0.05).


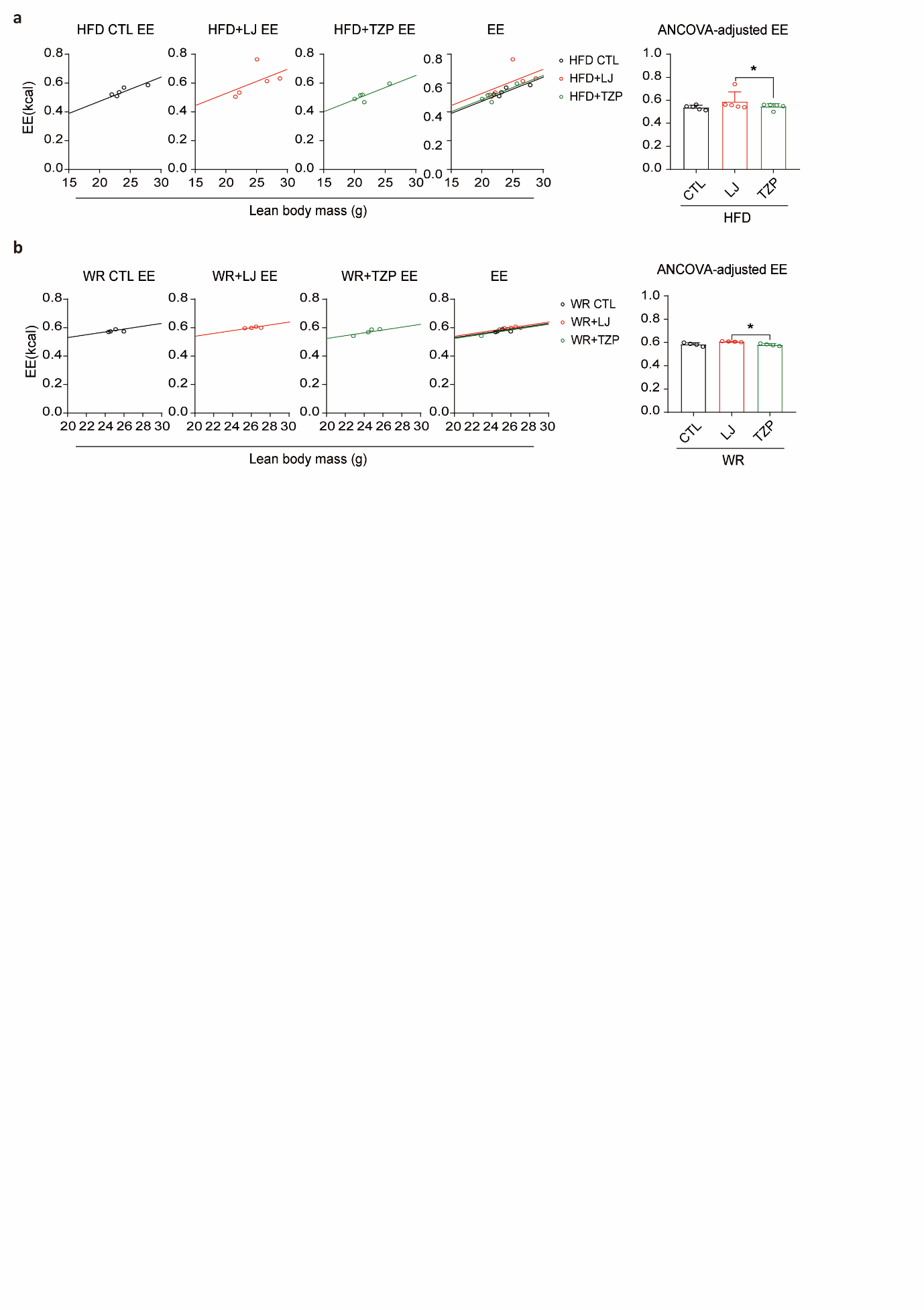


**Fig. S4 LJ-4378 treatment increases energy expenditure in HFD and WR groups.**

**a-b.** EE analyzed by ANCOVA with lean body mass as a covariate in HFD-fed mice and WR mice. Statistical significance was determined using ANCOVA analysis. Values are presented as mean ± SEM (^∗^*p* < 0.05).


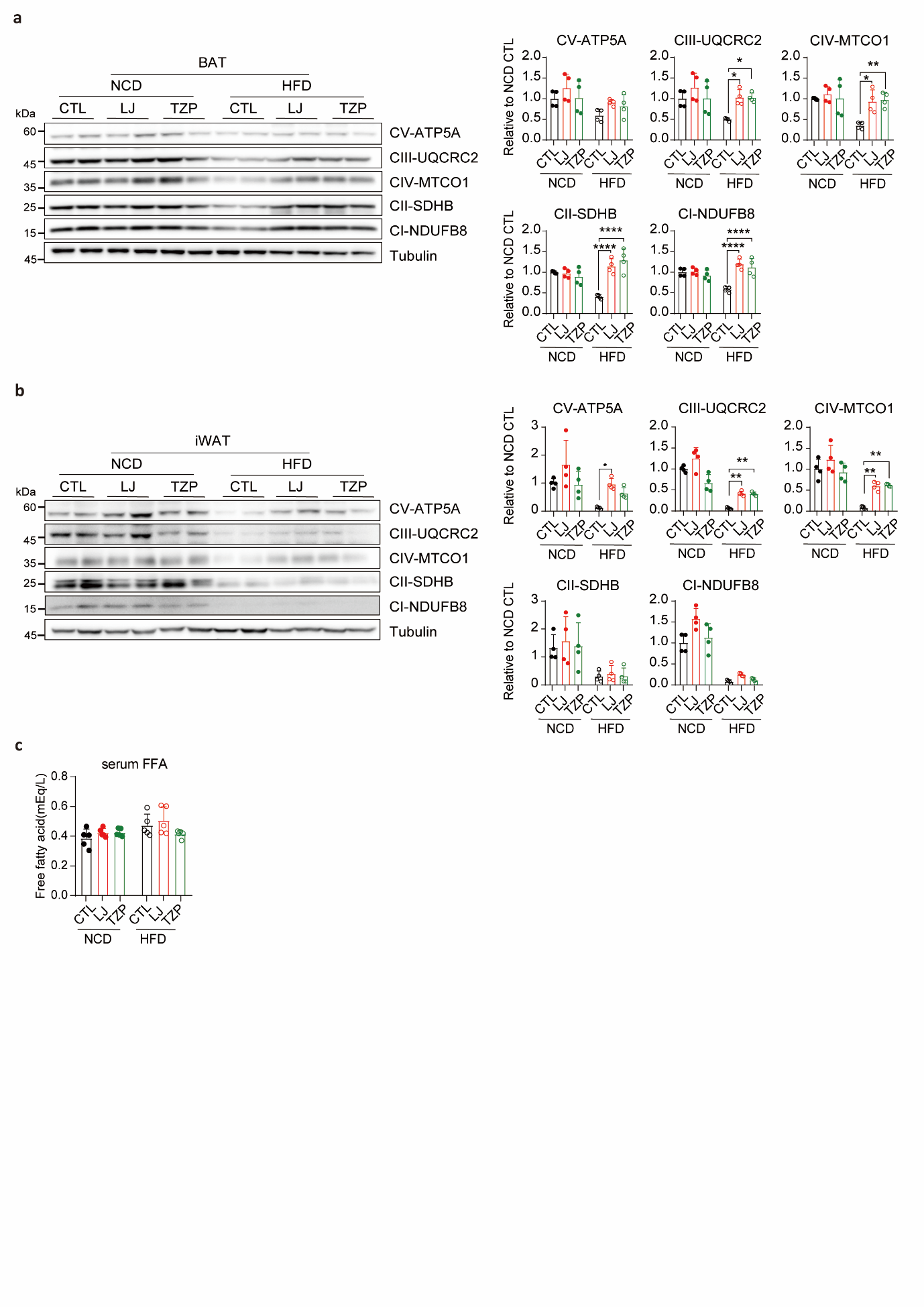


**Fig. S5 Effect of LJ-4378 and TZP on mitochondrial protein levels in adipose tissues and serum FFA levels.**

**a, b.** Western blot analysis of proteins involved in mitochondrial oxidative phosphorylation [ATP synthase complex 5 (ATP5A), ubiquinol-cytochrome-c reductase complex core protein 2 (UQCRC2), mitochondrially encoded cytochrome c oxidase I (MTCO1), succinate dehydrogenase iron-sulfur subunit (SDHB), NADH dehydrogenase ubiquinone 1 beta subcomplex subunit 8 (NDUFB8)] in BAT and iWAT of NCD- or HFD-fed mice treated with vehicle, LJ-4378, or TZP. n = 4. Significant effects of diet (ATP5A(BAT): *p* = 0.1287, UQCRC2(BAT): *p* = 0.0167, MTCO1(BAT): *p* = 0.0207, SDHB(BAT): *p* = 0.0003, NDUFB8(BAT): *p* = 0.0004 and ATP5A(iWAT): *p* = 0.0073, UQCRC2(iWAT): *p* = 0.0005, MTCO1(iWAT): *p* = 0.0045, SDHB(iWAT): *p* = 0.8186, NDUFB8(iWAT): *p* < 0.0001) and treatment (ATP5A(BAT): *p* = 0.0087, UQCRC2(BAT): *p* = 0.0305, MTCO1(BAT): *p* = 0.0116, SDHB(BAT): *p* = 0.0003, NDUFB8(BAT): *p* = 0.7714 and ATP5A(iWAT): *p* = 0.0018, UQCRC2(iWAT): *p* < 0.0001, MTCO1(iWAT): *p* < 0.0001, SDHB(iWAT): *p* = 0.0002, NDUFB8(iWAT): *p* = 0.0033) were observed. **c.** FFA levels in serum were analyzed. Significant effects of diet (*p* = 0.2244) and treatment (*p* = 0.0216) were observed. Statistical significance was determined using two-way ANOVA followed by Bonferroni post hoc test. Values are presented as mean ± SEM (^∗∗∗∗^*p* < 0.0001, ^∗∗^*p* < 0.01, ^∗^*p* < 0.05).


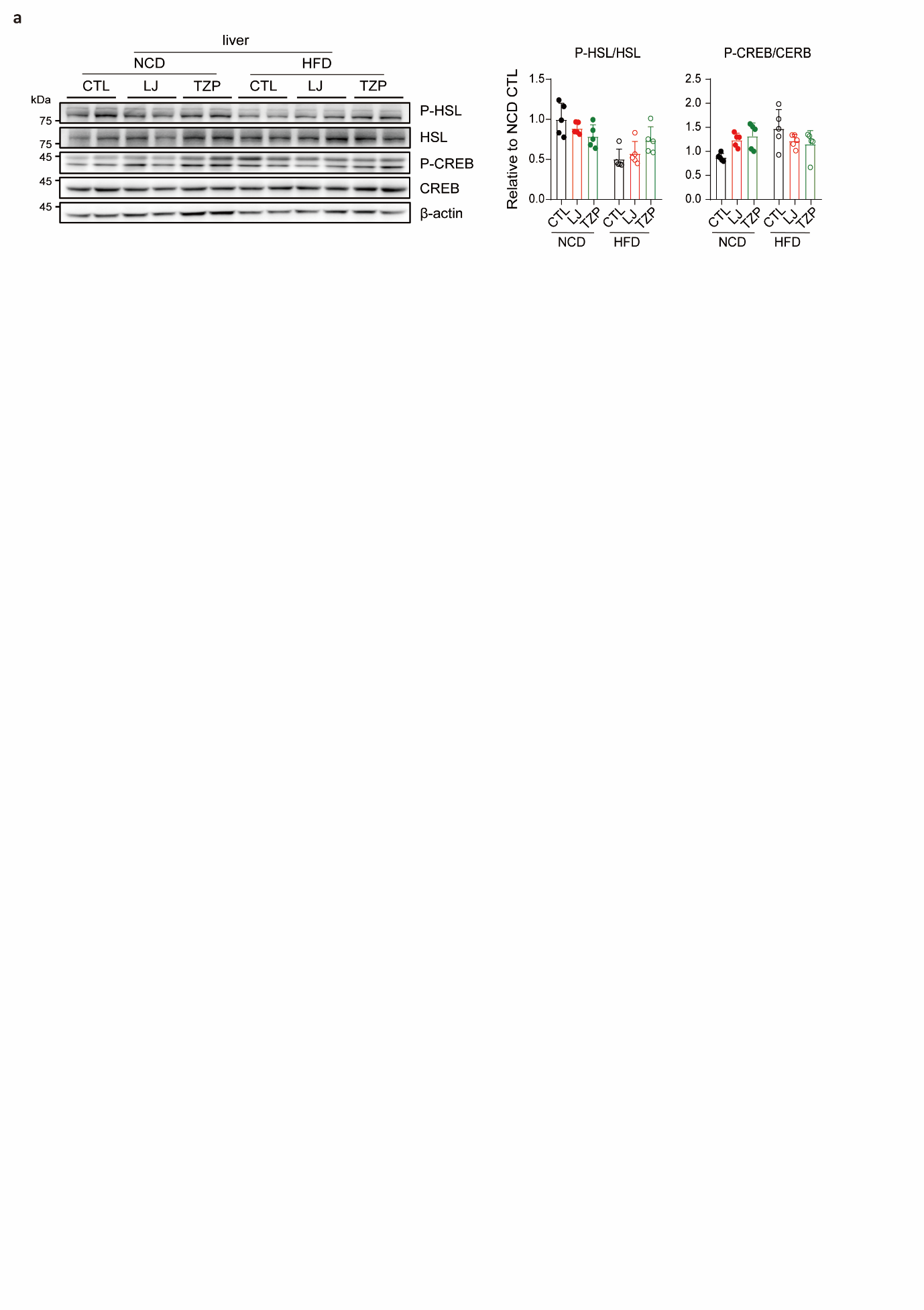


**Fig. S6 Effects of LJ-4378 and TZP treatment on PKA downstream protein phosphorylation in liver.**

Western blot analysis of P-HSL/HSL and P-CREB/CREB in liver. Significant effects of diet (P-HSL/HSL: *p* = 0.8883, P-CREB/CERB: *p* = 0.8296) and treatment (P-HSL/HSL: *p* < 0.0001, P-CREB/CERB: *p* = 0.1347) were observed. Statistical significance was determined using two-way ANOVA followed by Bonferroni post hoc test.


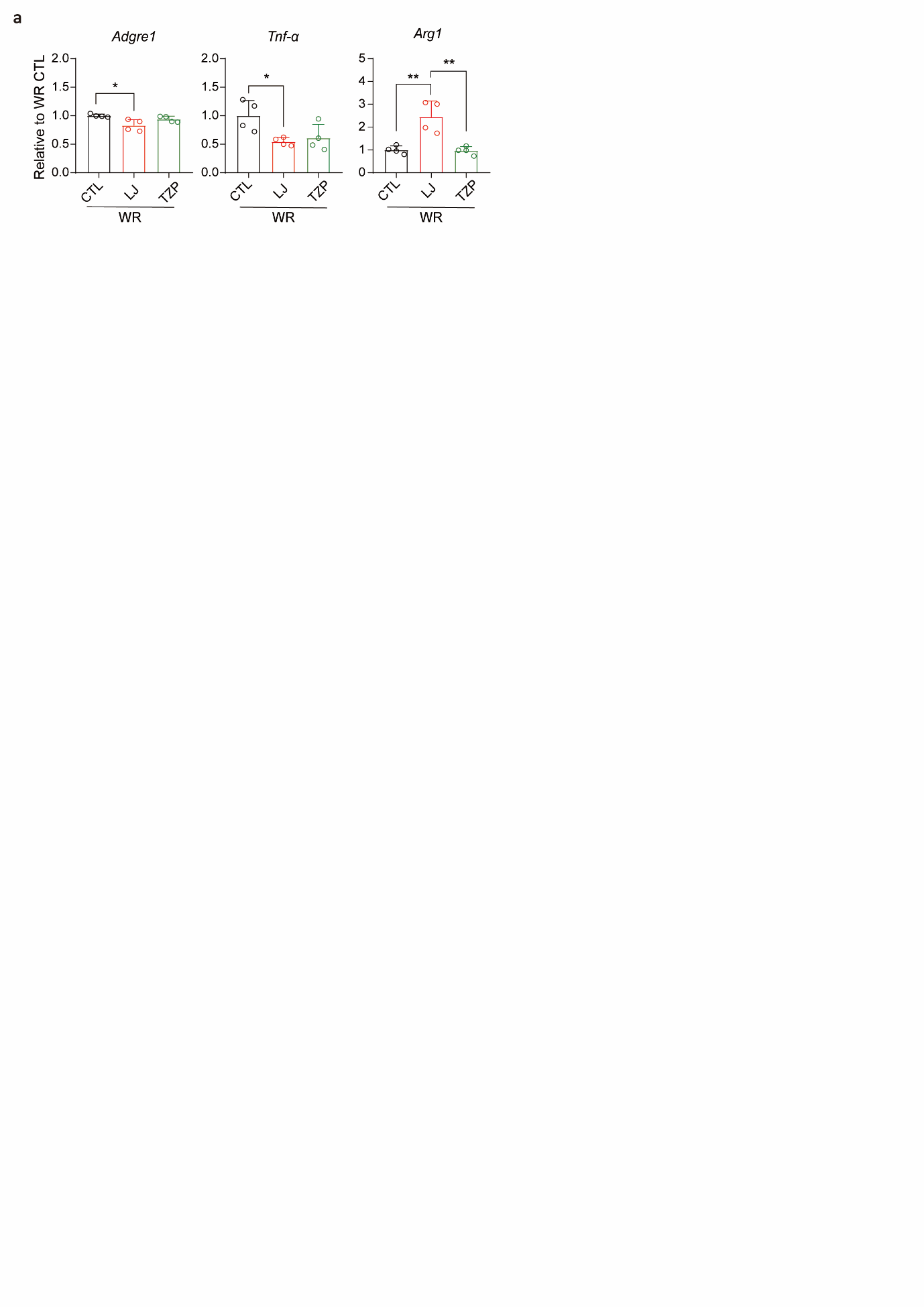


**Fig. S7 Sustained anti-inflammatory effects of LJ-4378 on adipose tissue after 4 weeks of discontinuation (WR groups)**

RT-qPCR analysis of *Adgre1*, *Tnf-α*, and *Arg1* gene expression in gWAT at 4 weeks of post-treatment cessation in the WR models. n = 4. Significant group effects (*Adgre1*: *p* = 0.0280, *Tnf-α*: *p* = 0.0194, *Arg1*: *p* = 0.0012) were observed. Statistical significance was determined using one-way ANOVA followed by Bonferroni post hoc test. Values are presented as mean ± SEM (^∗∗^ *p* < 0.01, ^∗^ *p* < 0.05).


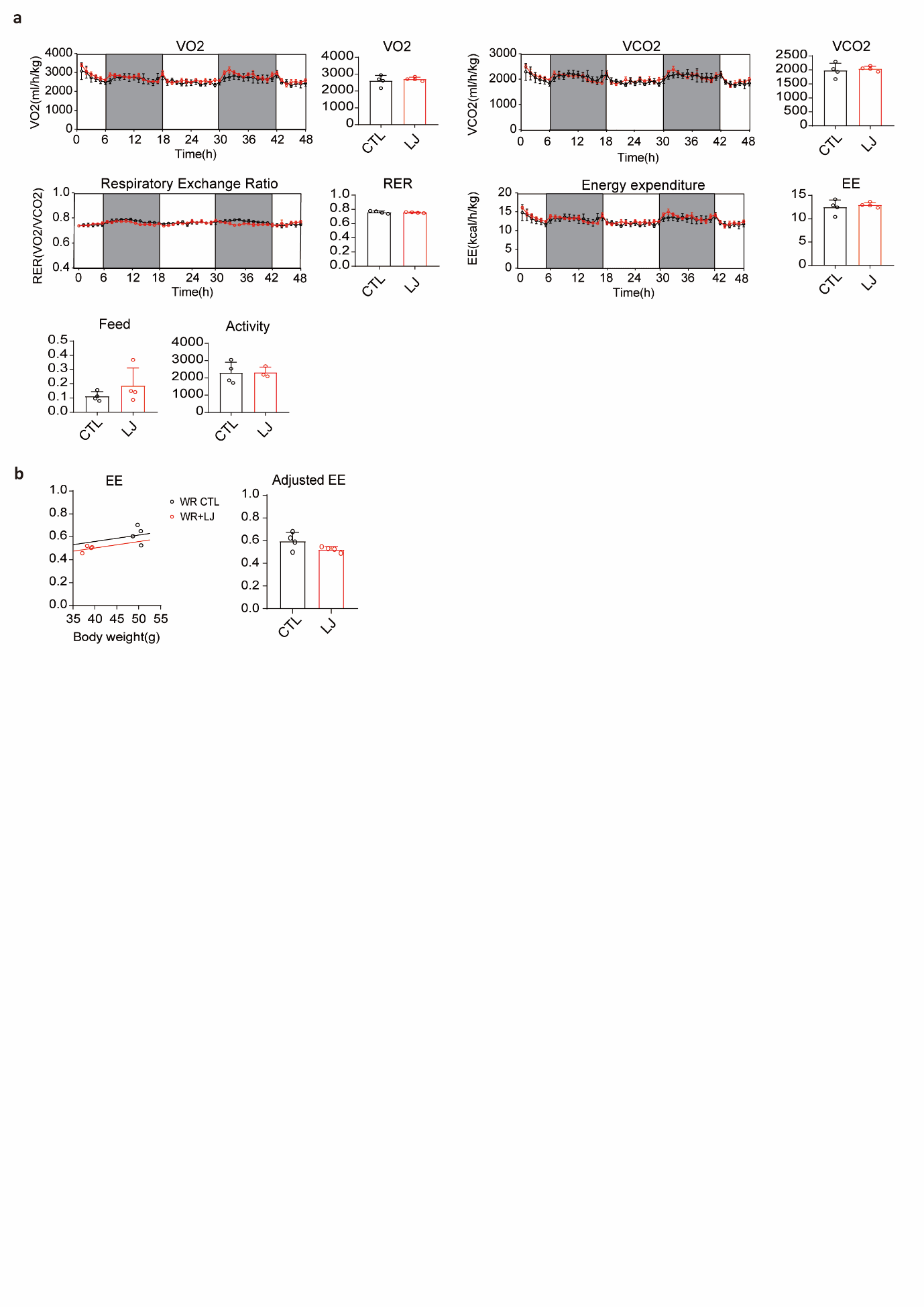


**Fig. S8 Indirect calorimetry analysis at 4 weeks post-treatment in WR mice treated with LJ-4378 treatment.**

**a.** Indirect calorimetry analysis at 4 weeks post-treatment in WR groups. n = 4. **b.** Energy expenditure was analyzed by ANCOVA with body mass as a covariate in WR mice. n = 4. Statistical significance was determined using an unpaired two-tailed *t*-test in **a,** and by an ANCOVA analysis **b**.


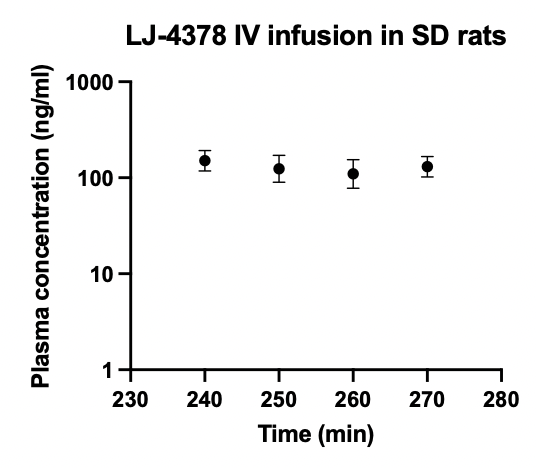


**Fig. S9 Plasma concentration of LJ-4378 after IV infusion in SD rats.**

From 240 to 270 min after the infusion, mean plasma concentration remained relatively constant (*p* = 0.525), indicating steady state levels of LJ-4378. n = 4. Statistical significance was determined using one-way ANOVA.


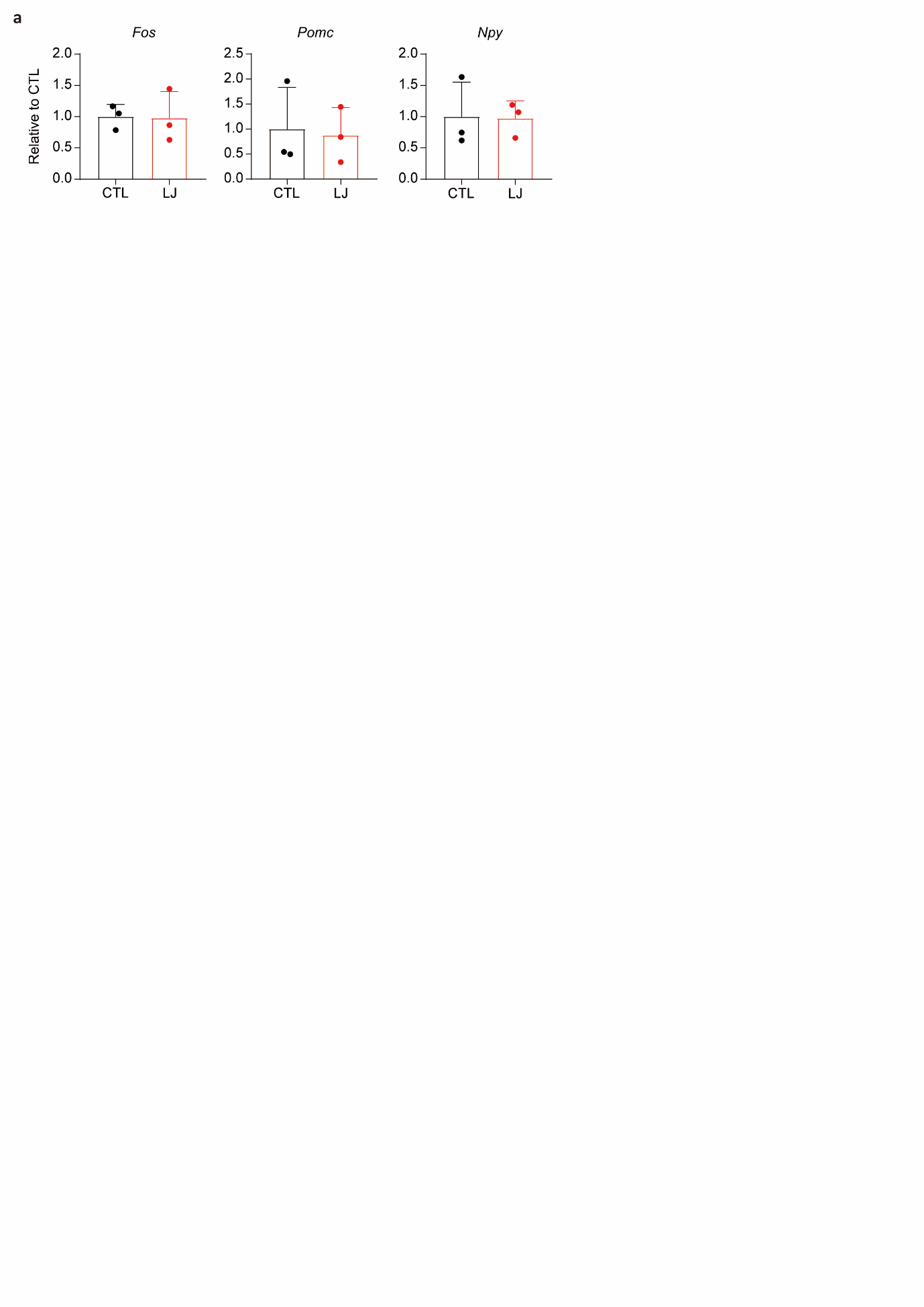


**Fig. S10. Effects of LJ-4378 treatment on hypothalamic gene expression.**

RT-qPCR analysis of Fos proto-oncogene (*Fos*), pro-opiomelanocortin-alpha (*Pomc*), and neuropeptide Y (*Npy*) in mouse hypothalamus 3h after vehicle or LJ-4378 (1mg/kg) treatment. n = 3. Statistical significance was determined using an unpaired two-tailed *t*-test.
